# Grape ASR Regulates Glucose Transport, Metabolism and Signaling

**DOI:** 10.3390/ijms23116194

**Published:** 2022-05-31

**Authors:** Jonathan Parrilla, Anna Medici, Cécile Gaillard, Jérémy Verbeke, Yves Gibon, Dominique Rolin, Maryse Laloi, Ruth R. Finkelstein, Rossitza Atanassova

**Affiliations:** 1 UMR CNRS 7267 Écologie et Biologie des Interactions, Équipe Sucres & Echanges Végétaux Environnement, Université de Poitiers, 3 Rue Jacques Fort, 86073 Poitiers, France; jonathan_parrilla@outlook.fr (J.P.); anna.medici@supagro.fr (A.M.); cecile.gaillard@univ-poitiers.fr (C.G.); jeremy.verbeke@uca.fr (J.V.); maryse.laloi@univ-poitiers.fr (M.L.); 2 Institut des Sciences des Plantes de Montpellier (IPSiM), UMR CNRS/INRAE/Institut Agro/Université de Montpellier, 2 Place Pierre Viala, 34000 Montpellier, France; 3 GReD-UMR CNRS 6293/INSERM U1103, CRBC, Faculté de Médecine, Université Clermont-Auvergne, 28 Place Henri Dunant, 63001 Clermont-Ferrand, France; 4 UMR 1332 Biologie du Fruit et Pathologie (BFP), INRA, Université de Bordeaux, 33882 Bordeaux, France; yves.gibon@inrae.fr (Y.G.); dominique.rollin@inrae.fr (D.R.); 5 Department of Molecular, Cellular, and Developmental Biology, University of California, Santa Barbara, CA 93106, USA; finkelst@ucsb.edu

**Keywords:** ASR, VvMSA overexpression/repression, VvHT1, grape embryogenic and non-embryogenic cells, glucose signaling, glucose absorption, sugar transporters, central metabolism, *Arabidopsis abi8* mutant, ABA/glucose sensitivity, germination

## Abstract

To decipher the mediator role of the grape Abscisic acid, Stress, Ripening (ASR) protein, VvMSA, in the pathways of glucose signaling through the regulation of its target, the promoter of hexose transporter VvHT1, we overexpressed and repressed *VvMSA* in embryogenic and non-embryogenic grapevine cells. The embryogenic cells with organized cell proliferation were chosen as an appropriate model for high sensitivity to the glucose signal, due to their very low intracellular glucose content and low glycolysis flux. In contrast, the non-embryogenic cells displaying anarchic cell proliferation, supported by high glycolysis flux and a partial switch to fermentation, appeared particularly sensitive to inhibitors of glucose metabolism. By using different glucose analogs to discriminate between distinct pathways of glucose signal transduction, we revealed VvMSA positioning as a transcriptional regulator of the glucose transporter gene *VvHT1* in glycolysis-dependent glucose signaling. The effects of both the overexpression and repression of VvMSA on glucose transport and metabolism via glycolysis were analyzed, and the results demonstrated its role as a mediator in the interplay of glucose metabolism, transport and signaling. The overexpression of VvMSA in the *Arabidopsis* mutant *abi8* provided evidence for its partial functional complementation by improving glucose absorption activity.

## 1. Introduction

Soluble sugars are key metabolites of plant central metabolism, and also structural units for cell wall and reserve polysaccharides, which constitute the major stock of carbon storage. The transport of these primary photoassimilates over long distance is crucial for the establishment of source- and sink-organ relationships during plant development, and responses to abiotic and biotic constraints. In parallel, their transport over short distance between neighboring cells and their partitioning between different subcellular compartments implies an important role in maintaining osmotic pressure, and thereby cell turgidity and expansion. Some sugars are well-established metabolic signals (glucose, sucrose, fructose, trehalose/trehalose-6-P) [1,2,3,4,5,6], and their interplay with other metabolic, hormonal and environmental signals is involved in the regulation of cell proliferation and differentiation, plant growth and development [1,7,8,9]. Abscisic acid, Stress, Ripening-induced proteins (ASR) have been identified and characterized as regulated at the interface of glucose and ABA signaling pathways, at the transitions between development stages and in the responses to environmental cues relying on shifts of cell/organ sugar status [10,11,12,13,14,15,16,17,18].

### 1.1. ASR Proteins: Emergence, Molecular Features and Biological Functions

Since the discovery of the first ASR in Tomato [19], these proteins have been identified in a plethora of plant species. After having applied approaches of evolutionary genomics to 57 sequenced genomes, from Algae to Angiosperms, we identified 165 proteins belonging to 35 species, whose phylogenic tree is presented in Figure S1. It should be noted that we did not find any ASRs in Algae, Bryophytes and Lycophytes, whereas several ASRs have been identified in *Pinus taeda*, confirming those already described [20,21] and providing evidence for the early emergence of the ASRs in the common ancestor of Gymnosperms and Angiosperms. In addition, our phylogenic analysis confirmed the absence of *ASR* genes in the genomes of the *Brassicaceae* family, as that has already been suggested for *Arabidopsis thaliana* and the Arabidopsis-related species *Thellungiella halophilla* [22] and the crucifer heavy metal hyperaccumulator *Thlaspi caerulescens* [23]. When we compare the evolutionary history of ASRs with that of terrestrial plants, it is interesting to note that the emergence of ASRs is in accordance with the restriction of plants’ desiccation tolerance from the whole organism to some organs (seeds, pollen).

The description of several ASRs as intrinsically disordered proteins [24,25,26,27,28] perfectly matches with their ability to interact as chaperones with macromolecules, such as nucleic acids and enzymes [29,30,31]. ASR proteins can also act as transcription regulators able to recognize and bind to specific promoter sequences [32,33,34,35,36], as well as to form homodimers and heterodimers with other transcription factors [37,38,39,40]. All these molecular features in combination with their subcellular distribution, nuclear and cytosolic [32,33,34,35,41], contribute to their physiological functions at the transitions between plant developmental stages [18,19,32,42] and in the plant responses to abiotic and biotic constraints [12,15,16,36,43,44,45,46,47,48,49,50]. Furthermore, the deciphering of ASRs’ plausible involvement in the epigenetic mechanisms throughout their impact on microRNA expression, chromatin modifiers, and histone marks [27,51] may suggest a real biological role in the priming of plant responses to environmental cues.

### 1.2. Involvement of ASR Proteins in Glucose Signaling, Metabolism, and Transport

The results of the glucose signaling studies support three plausible transduction pathways: one dependent on the sensor function of hexokinase 1 (HXK1); a second one dependent on the catalytic function and independent of the sensor function of HXK1, referred to as the glycolysis dependent pathway; a third one independent of HXK1 and potentially initiated by a plasma membrane sensor [52]. It has been demonstrated that the metabolic activity of AtHXK1 is uncoupled from its role in signaling, and vice versa, the glucose-sensing function is found to be independent of its catalytic activity [53]. In Grape, we have demonstrated for the first time the involvement of an ASR, VvMSA (*Vitis vinifera* Maturation, Stress, Abscisic acid-induced), in the transcriptional regulation of the hexose transporter gene *VvHT1* (*Vitis vinifera Hexose Transporter 1*) by its proper substrate, glucose [32]. Moreover, we deciphered the fine tuning of *VvMSA* gene expression at the crosstalk of glucose and ABA signaling, and built a model of this metabolic and hormonal interplay throughout the HXK1 and SnRK1 (Sucrose non-fermenting Related Kinase 1) interaction [54]. It has been reported that compared to wild-type plants, transgenic potatoes overexpressing tomato *ASR1* increase their tuber number and reduce their glucose content, in a concomitant manner with expression inhibition of the hexose transporter homolog *LeHT2* [55]. In the same context, the overexpression of maize *ASR1* has been demonstrated to improve the yield and to modulate the expression of several genes and proteins involved in fatty acid metabolism [56]. The latter observation is specific for plants; after germination, the lipids stored in seeds are subsequently transformed in peroxisome and mitochondrion to malate, which after conversion to phosphoenolpyruvate in cytosol is metabolized via the gluconeogenic pathway to sucrose, in order to be reallocated by the phloem to the sink-organs. Furthermore, it has been reported that the constitutive expression of tomato *ASR1* in antisense orientation in tobacco induces a marked dwarf genotype, corresponding to reduced ABA content, and inversely, to elevated glucose content in leaves [57]. That accumulation of glucose in leaves is concomitant with the repression of one hexose transporter, *NtHT1,* and the sucrose transporter *NtSUT2*. Similarly, the gene expression of HXK1 and SnRK1 (two protein-kinases involved in sugar signaling) displays an inverse relationship: when the first one is induced, the second one is inhibited. The effect of increased glucose content under *ASR1* silencing perfectly corresponds to the HXK1 function as a cytosolic sensor responsible for repression of photosynthetic genes [57]. These overall data strongly imply the involvement of ASRs in the regulation of sugar metabolism and carbon allocation, as proteins acting at the crosstalk of glucose and hormonal signaling.

### 1.3. Regulation of Sugar Transporters by Their Own Substrates

Transcriptomic analyses have demonstrated that several genes of monosaccharide transporters [58,59,60,61,62] and disaccharide transporters [63,64,65,66,67,68] are regulated by sugars. In beans (*Vicia faba*), it has been reported that glucose and sucrose inhibit *VfSUT1* gene expression at high concentration (150 mM), while this effect is not observed at low sugar concentration (10 mM) [63]. However, the signaling pathways involved in the regulation of sugar transporters at the protein level remain insufficiently elucidated. In the latter regard, some early studies have suggested the involvement of HXK1 in the activity regulation of certain monosaccharides transporters [60,69]. A more recent work has reported that the *Arabidopsis Sugar Transporter1* (*AtSTP1)* is induced by sugar depletion and is inhibited by glucose in an HXK1-independent manner. Moreover, the glucose inhibition of *AtSTP1* requires previous phosphorylation of glucose [70]. As the overexpression of the catalytic subunit of SnRK1, AtKIN10, has been reported to affect the expression of the *AtSTP1* gene [70], SnRK1 should also regulate sugar transporters. However, the same authors did not observe any marked effect of AtKIN10 overexpression on the inhibition of *AtSTP1* by glucose. The expression of *VvHT1* is mediated by VvMSA [32], and the expression of *VvMSA* itself is controlled by glucose [54], which implies the role of grape ASRs in the transcriptional regulation of the hexose transporter VvHT1 by its proper substrate, glucose. In this regard, other studies also corroborate the idea that the ASR proteins could participate in the regulation of sugar transporters by glucose signaling [55,57].

In this study, we explored the biological functions of grape ASR, VvMSA, through its overexpression or repression in genetically transformed grape cell lines of both types, embryogenic (EC) and non-embryogenic (NEC). As previously described by us, the embryogenic cells have been characterized by organized cell proliferation, low intracellular glucose content and low glycolysis flux, while the non-embryogenic cells displayed anarchic cell proliferation, supported by high glucose content and high glycolysis flux [71]. In these well-characterized cell cultures, we deciphered VvMSA positioning as a transcriptional regulator of the glucose transporter gene *VvHT1* in glucose signaling, using different glucose analogs to discriminate between distinct pathways of glucose signal transduction. Furthermore, the impacts of VvMSA repression on glucose transport and metabolism via glycolysis were analyzed and its role as mediator in the interplay of glucose metabolism, transport and signaling was demonstrated. We found that overexpression of VvMSA partially restored glucose and ABA sensitivity of seed germination in the Arabidopsis mutant *abi8*.

## 2. Results

### 2.1. Analysis of VvMSA Overexpression and Silencing in Grape Cells

**Figure 1:**
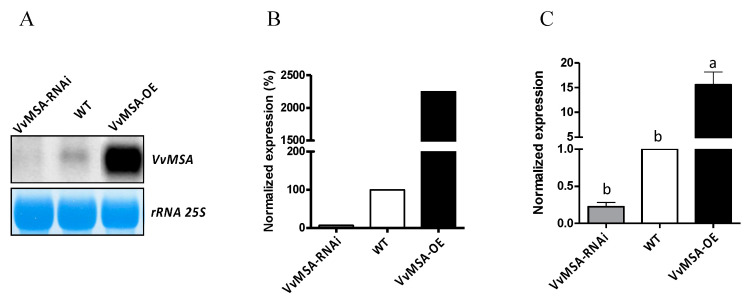
Analysis of the *VvMSA* expression in WT, VvMSA-OE and VvMSA-RNAi embryogenic cells. (**A**) Northern blot obtained after hybridization of radiolabeled probe corresponding to cDNA of *VvMSA*. Lower panel shows methylene blue staining of the filter. (**B**) Quantitation of the Northern blot signals presented as percentage of *VvMSA* expression of the transgenic lines reported to that of the WT line (considered as 100%). (**C**) RT-qPCR: the relative expression of the VvMSA genes is calculated by the ΔΔCt method using *VvGAPDH* as a reference gene and *VvMSA* in WT as a control gene. The histograms show the mean of three independent experiments performed on 8-day-old cells (±SEM). The letters denote the groups that were found significantly different by ANOVA and Fisher LSD test (*p* < 0.05).

**Figure 2:**
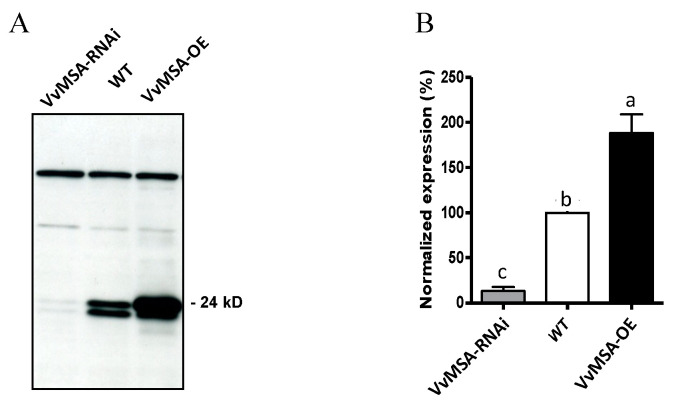
Western blot analysis of the *VvMSA* expression in WT, VvMSA-OE and VvMSA-RNAi cell suspensions. (**A**) Immune detection of VvMSA proteins separated by SDS-PAGE of 20 µg total protein extract of each of the cell suspensions and transferred onto nitrocellulose membrane. The prominent higher MW non-specific bands with even intensity, that are immunorevealed by the polyclonal anti-VvMSA antibody, serve as a loading control. (**B**) Signal quantitation using ImageJ, v.1.4. The *VvMSA* expression of the transgenic lines is presented as a percentage of that of the WT (considered as 100%). The histograms show the mean of three independent experiments. The letters denote the groups that were found significantly different by ANOVA and Fisher LSD test (*p* < 0.05).

After confirming the integration of the *p35S::VvMSA* and *p35S::VvMSA-RNAi* constructs in the grape embryogenic cell 41B genome, the gene overexpression and repression at day 8 after subculture were studied by Northern blot (Figure 1A,B) and RT-qPCR (Figure 1C). The level of *VvMSA* expression in the wild-type cells (WT) was sharply induced in *p35S::VvMSA* (VvMSA-OE), while it was strongly reduced in *p35S::VvMSA-RNAi* (VvMSA-RNAi) cells. The transgene impact was also studied at the protein level by Western blot (Figure 2A,B), using a custom made VvMSA specific antibody [42].

As shown in Figure 2A, the two grape ASR allelic forms, complete and deleted (lacking five amino acids, without frame shift [38]), were equally present in the WT, almost totally absent in the VvMSA-RNAi silenced cells, while the complete form was highly overexpressed in the VvMSA-OE cells.

**Figure 3:**
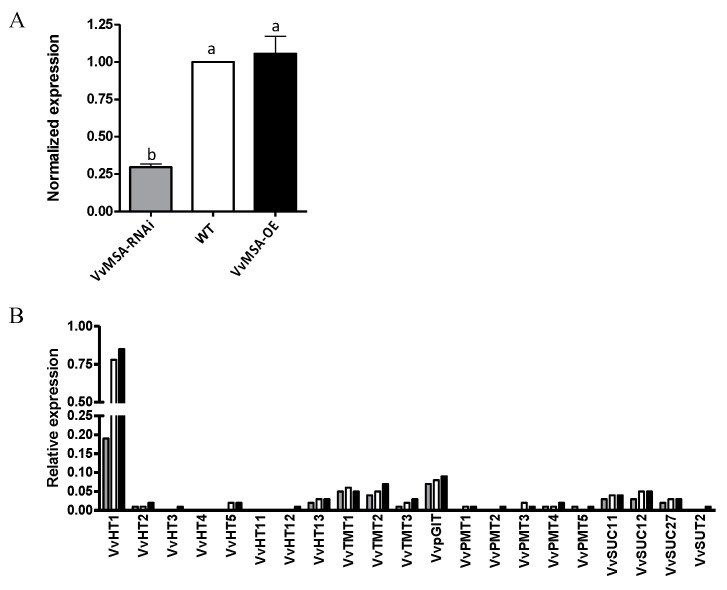
Expression of sugar transporters in WT, VvMSA-OE and VvMSA-RNAi embryogenic cells (EC) after 8 days of culture. (**A**) RT-qPCR analysis of the *VvHT1* expression. The relative expression of the *VvHT1* gene is calculated by the ΔΔCt method using *VvGAPDH* as a reference gene and *VvMSA* in WT as a control gene. The histograms show the mean of three independent experiments performed on 8-day-old cells (±SEM). The letters denote the groups that were found significantly different by ANOVA and Fisher LSD test (*p* < 0.05). (**B**) Macroarray analysis of sugar transporter expression. The histograms show the mean of two independent experiments performed on 8-day-old cells. Expression values are normalized using four housekeeping genes *VvGAPDH*, *VvActin*, *VvEF1a*, *VvEF1γ*.

These results raised the biological question regarding the overexpression/silencing impact of VvMSA as a transcription factor on its target gene encoding the hexose transporter VvHT1. As demonstrated by RT-qPCR analysis (Figure 3A), the expression level of *VvHT1* remained almost at the same level between the WT and the VvMSA-OE cells. This implies that endogenous VvMSA is sufficient for maximal *VvHT1* expression in OE cells, and additional response may be limited by a binding partner. Inversely, VvHT1 expression strongly decreased in the VvMSA-RNAi cells. This sharp reduction was estimated at about 75% and perfectly confirmed the role of VvMSA protein in *VvHT1* gene expression regulation.

To explore the impact of grape ASR silencing beyond its target *VvHT1* gene, a thematic macroarray representative for the grape subfamilies of hexose transporters VvHTs, tonoplast monosaccharide transporters TMT, polyol monosaccharide transporters PMT, plastid glucose transporters VvpGlT and sucrose carriers VvSUC, was hybridized with the mRNAs extracted at day 8 from each of the three embryogenic cell cultures (Figure 3B). Moreover, even though the *VvHT1* expression in VvMSA-RNAi cells was strongly inhibited, it remained the largely dominant glucose transporter gene under heterotrophic conditions as compared to the other studied sugar transporter genes. The obtained results clearly demonstrated that only *VvHT1* was strongly regulated by the grape ASR, and consequently, none of the other studied sugar transporter genes were able to compensate for the important reduction of *VvHT1* expression in the grape embryogenic cells.

**Figure 4:**
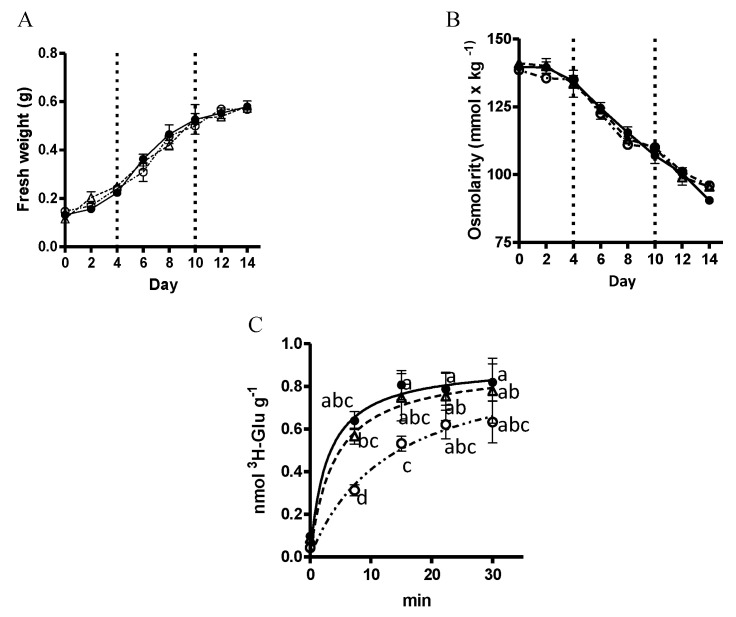
(**A**) Cell growth kinetics of WT, VvMSA-OE and VvMSA-RNAi embryogenic cells (EC). The cell proliferation is expressed as a function of the cell fresh weight. ●-WT; △-VvMSA-OE; ◯-VvMSA-RNAi. (**B**) Osmolarity of the culture media of the WT, VvMSA-OE and VvMSA-RNAi cell lines. The dots represent the mean values (±SEM) that were obtained after measurement of 10 µL of medium by using Wescor VAPRO vapor pressure osmometer, model 5520. (**C**) Absorption of D-[^3^H]-glucose measured in WT, VvMSA-OE and VvMSA-RNAi cells grown at 0.2 mM glucose concentration. The D-[^3^H]-glucose absorption was reported to the dry weight of the cells. The nonlinear regression curves in (**A**–**C**) were generated from the mean values (±SEM) of three independent experiments and analysis by ANOVA and Fisher LSD test (*p* < 0.05). No significant differences were found between the groups that were compared either by fresh weight (**A**) or osmolarity (**B**), while the comparison by absorption of D-[^3^H]-glucose allowed us to discriminate between the groups (**C**). The letters on the dots in (**C**) indicate the level of significant difference between the groups at four time-points of 0, 10, 20 and 30 min.

The next issue concerned the physiological effect of *VvMSA* silencing and reduced *VvHT1* expression in terms of glucose absorption activity. As a first step, the absence of any phenotypic difference between the three types of embryogenic cells (WT, VvMSA-OE and VvMSA-RNAi) was confirmed by the perfect matching of their growth curves throughout the fourteen days of subculture period (Figure 4A). Overexpression and repression of *VvMSA* did not modify the osmolarity of the culture media of the three cell lines, whose greatest decrease from 140 mmol·kg^−1^ to 112 mmol·kg^−1^ corresponded to the exponential phase of cell growth (Figure 4B). Furthermore, while the comparison of the glucose absorption in these three cell suspensions revealed an absence of notable differences between the WT and VvMSA-OE cells, the glucose transport activity was significantly inhibited in the VvMSA-RNAi cells, consistent with the *VvHT1* transcript data (Figure 4C).

### 2.2. Stability of VvMSA Silencing and VvHT1 Expression Evolution

An important observation concerned the disappearance of *VvHT1* expression inhibition in VvMSA-RNAi cells at a long term (Figure S2), while the repression of *VvMSA* was perfectly confirmed in embryogenic cells (EC), the regenerated vitroplants and non-embryogenic cells (NEC) (Figure S3). To highlight this discrepancy between inhibition of *VvHT1* expression by the *VvMSA* repression observed early after the transformation of EC cells with the VvMSA-RNAi construct, we performed in parallel two new independent transformations with the same VvMSA-RNAi construct and proceeded to the profiling of both concerned genes during and beyond the time period of culture stabilization.

The results perfectly confirmed the efficiency of VvMSA-RNAi transformation and the causal relationship between *VvMSA* repression and *VvHT1*-expression inhibition (Figure S4). The *VvMSA* repression was progressively imposed due to the selection of the VvMSA-RNAi transformed cells and became significant after four months, but without a very strong impact on *VvHT1* expression. After six months, the *VvMSA* repression was definitely stabilized and produced significantly inhibited *VvHT1* expression. Both transformed cell cultures exhibited the same level of *VvMSA* repression and a sustained *VvHT1* inhibition between the sixth and the eighth month. However, after eight months and despite the stability of VvMSA repression in all subcultures, the expression of *VvHT1* was progressively restored, as observed for the first genetic transformation (Figure S2).

### 2.3. Establishment of Treatment Conditions with Glucose and Its Analogs

Taking advantage of our recent demonstration of the differences in the glycolytic metabolism of embryogenic and non-embryogenic grape cells [71], we decided to study the positioning of the VvMSA/*VvHT1* interaction in the different pathways of the glucose signaling. The analysis of grape *ASR* gene expression in embryogenic and non-embryogenic cells clearly confirmed the overexpression and the silencing of *VvMSA* gene compared to the wild type, and thus allowed us to compare six cellular models. (Figure S4).

In order to study the positioning of VvMSA impact on *VvHT1* expression in the three well-identified pathways of glucose signaling, we applied a pharmacological approach using D-glucose and its analogs: D-mannose, 2-deoxyglucose (2DOG), 3-*O*-methylglucose (3OMG) and mannitol as osmotic pressure control, as well as the inhibitor of the hexokinase catalytic activity—the mannoheptulose. In this way, we studied, in parallel with *VvHT1*, the gene encoding VvHT5 hexose transporter, whose expression appeared also to be regulated under biotic and abiotic stresses.

**Figure 5:**
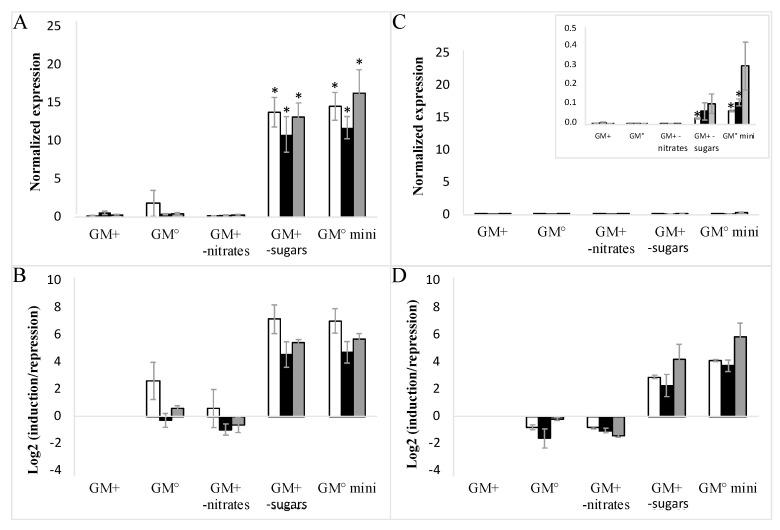
Influence of cell culture media composition on the expression of *VvHT1* and *VvHT5* in embryogenic cells (EC). Complete medium (GM^+^), medium without auxins (GM°), medium without nitrates (GM^+^ w/o nitrates), medium without maltose and glycerol (GM^+^ w/o sugars), medium without auxins, nitrates, maltose and glycerol (GM° mini). □-WT; ■-VvMSA-OE; ■-VvMSA-RNAi. The gene expression of *VvHT1* (**A**,**B**) and *VvHT5* (**C**,**D**) is normalized to that of the housekeeping gene *VvActin* reported to the control condition used to calculate the ratio of induction/repression. Normalized expression (**A**,**C**) and induction/repression (**B**,**D**) are calculated by the methods of ΔCt and 2^−ΔΔCT^ respectively. The presented results correspond to the mean values of three independent experiments (±SEM). The asterisks denote the groups that were found significantly different by Kruskal–Wallis test (* *p* < 0.05).

To avoid mutual interference between sugar and nitrate signaling, all cell lines of EC and NEC type were subcultured in minimal media depleted of sugars and nitrates (Figure 5A–D). The profiles of both *VvHT1* and *VvHT5* genes in the presence or absence of nitrates unambiguously demonstrated that the sugar depletion most strongly induced *VvHT1* and *VvHT5* expression in the EC wild-type suspension. In agreement with the macroarray analysis of hexose transporter genes, the *VvHT1* demonstrated a remarkably higher expression than that of *VvHT5* (Figure 3B).

**Figure 6:**
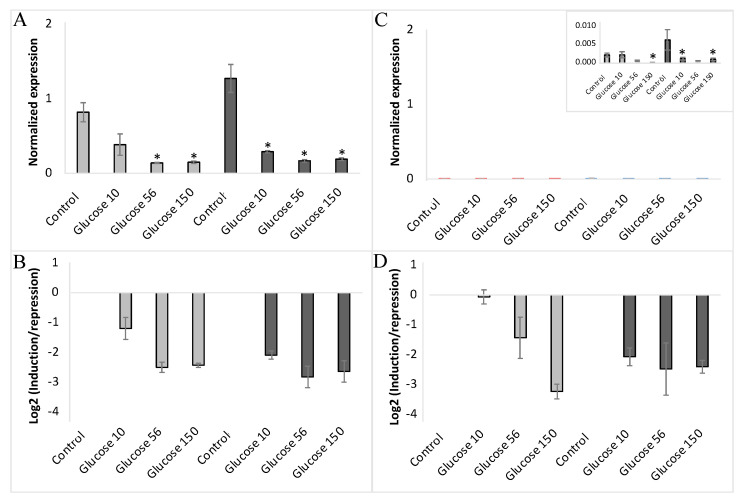
Expression of *VvHT1* and *VvHT5* in non-embryogenic cells (NEC) of wild-type cultured at three concentrations of glucose (10 mM, 56 mM and 150 mM) either in presence (■) or absence (■) of nitrates. “Glucose10”-10 mM; “Glucose56”-56 mM; “Glucose150”-150 mM. The gene expression of *VvHT1* (**A**,**B**) and *VvHT5* (**C**,**D**) is normalized to that of the housekeeping gene *VvActin* reported to the control condition used to calculate the ratio of induction/repression. Normalized expression (**A**,**C**) and induction/repression (**B**,**D**) are calculated by the methods of ΔCt and 2^−ΔΔCT^, respectively. The presented results correspond to the mean values of three independent experiments (±SEM). The asterisks denote the groups that were found significantly different by Kruskal–Wallis test (* *p* < 0.05).

To further explore the glucose regulation of *VvHT1* and *VvHT5* expression, we analyzed the effects of 10 mM, 56 mM and 150 mM glucose in culture media supplemented or depleted for nitrates (Figure 6A–D). The repression of both monosaccharide transporter genes by 10 mM glucose was strongly enhanced by nitrate depletion, while the impacts at 56 mM and 150 mM glucose concentration appeared to be slightly improved in the presence or absence of nitrates, albeit without any significant difference between them. These data demonstrated that 10 mM glucose, a concentration corresponding to the higher glucose concentration throughout the growth cycle of embryogenic cells [71], is sufficient to explore the expression regulation of the two hexose transporter genes in the glucose signaling pathways.

### 2.4. Positioning of VvMSA/VvHT1 Interaction in the Glucose Signaling Pathways

**Figure 7:**
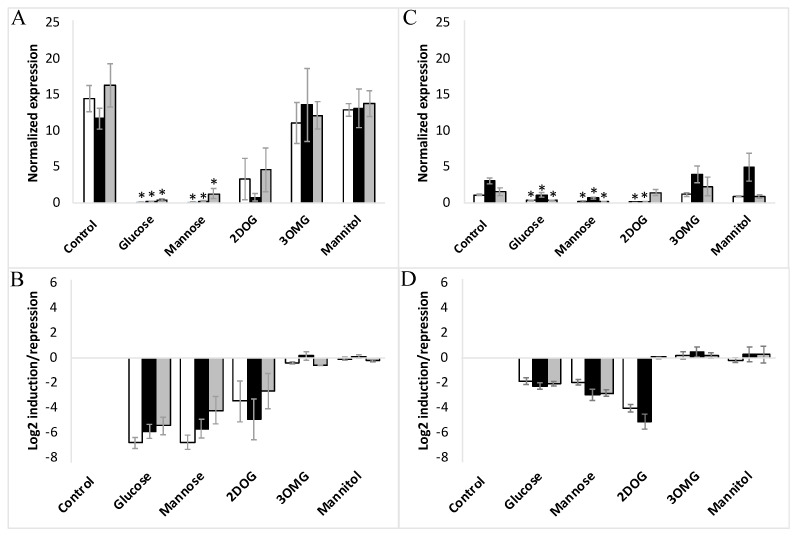
Expression of *VvHT1* in embryogenic cells (**A**,**B**) and non-embryogenic cells (**C**,**D**) in response to different treatments with glucose, mannose, 3OMG, mannitol (all applied at 10 mM concentration), and 2DOG (0.9 mM). □-WT; ■-VvMSA-OE; ■-VvMSA-RNAi. The embryogenic cells and the non-embryogenic cells were collected after 8 days of subculture and incubated for additional 24 h in their respective minimal media, i.e., GM° minimum (EC) and Gb minimum (NEC). Sugar effectors were added afterwards and the treatment of EC was extended for 6 h, while that of NEC for 24 h. The gene expression of *VvHT1* is normalized to that of the housekeeping gene *VvActin* and reported to the control condition used to calculate the ratio of induction/repression. Normalized expression (**A**,**C**) and induction/repression (**B**,**D**) are calculated by the methods of ΔCt and 2^−ΔΔCT^, respectively. The presented results correspond to the mean values of three independent biological experiments (±SEM). The asterisks denote the groups that were found significantly different by Kruskal–Wallis test (* *p* < 0.05).

On day 8 of the subculture, corresponding to the exponential phase of cell growth, embryogenic and non-embryogenic cells (WT, VvMSA-OE and VvMSA-RNAi) were transferred after washing into minimal media depleted of nitrates and sugars. After 24 h of culture of embryogenic and non-embryogenic cell lines under such nutrient deficiency, the hexokinase inhibitor mannoheptulose, glucose, or its analogs (mannose, 3OMG and mannitol) were added at physiological concentrations of 10 mM each (except 2DOG that was applied at 0.9 mM). The expression of *VvHT1* and *VvHT5* was first normalized to that of the reference gene *VvActin*, and then reported to the respective gene expression in the corresponding minimal medium as control conditions (Figure 7A–D and Figure S5).

It is noteworthy, that in the above-mentioned control conditions, the *VvHT1* expression level was at least ten-fold higher than that of *VvHT5*. The administration of 10 mM glucose imposed a very strong inhibition of *VvHT1*, which was 60-fold for EC and 4-fold for NEC, respectively (Figure 7A–D). However, the simultaneous treatment with 10 mM glucose and 10 mM mannoheptulose (presumed as HXK inhibitor) repressed *VvHT1* expression in the same manner as the treatment with the glucose alone (Figure S5). The administration of mannoheptulose from two different providers (Glycoteam, Hamburg, Germany; Sigma Aldrich, St. Louis, MO, USA) did not allow to abrogate the repression of *VvHT1* caused by the glucose.

**Figure 8:**
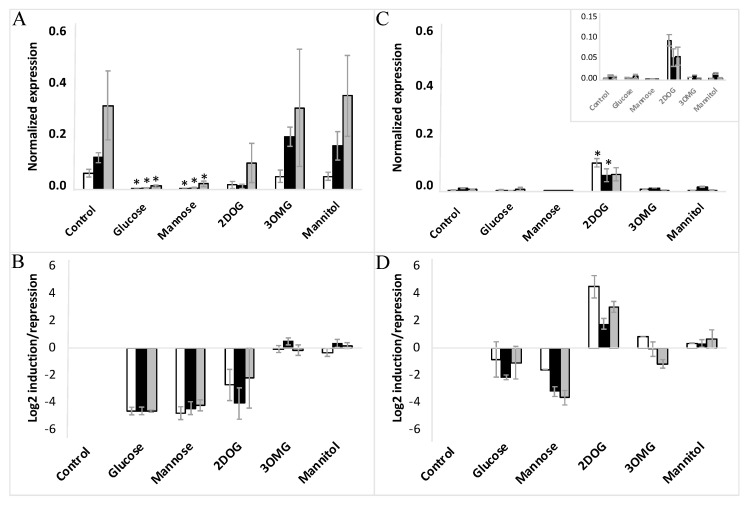
Expression of *VvHT5* in embryogenic cells (**A**,**B**) and non-embryogenic cells (**C**,**D**) in response to different treatments with glucose, mannose, 3OMG, mannitol (all applied at 10 mM concentration), and 2DOG (0.9 mM). □-WT; ■-VvMSA-OE; ■-VvMSA-RNAi. The embryogenic cells and the non-embryogenic cells were collected after 8 days of subculture and incubated for additional 24 h in their respective minimal media, i.e., GM° minimum (EC) and Gb minimum (NEC). Sugar effectors were added afterwards and the treatment of EC was extended for 6 h, while that of NEC for 24 h. The gene expression of *VvHT1* is normalized to that of the housekeeping gene *VvActin* and reported to the control condition used to calculate the ratio of induction/repression. Normalized expression (**A**,**B**) and induction/repression (**C**,**D**) are calculated by the methods of ΔCt and 2^−ΔΔCT^, respectively. The presented results correspond to the mean values of three independent biological experiments (±SEM). The asterisks denote the groups that were found significantly different by Kruskal–Wallis test (* *p* < 0.05).

Concerning *VvHT5*, its expression was reduced significantly by glucose (>16-fold) in the EC cells, but demonstrated just propensity for inhibition in the NEC cells, where its transcript level remained too weak under control conditions (Figure 8A–D). Mannose is known to be well transported into the cell and phosphorylated by HXK, but is a poorly metabolized analogue of glucose. This effector produced nearly the same reduction of *VvHT1* expression as glucose in the three cell lines of each EC and NEC type. The inhibitory effect of mannose on *VvHT5* expression was also similar to that of glucose on all three EC lines, but appeared more pronounced on NEC cells.

Keeping in mind that 3OMG is imported by the cell but negligibly phosphorylated by HXK, it is noteworthy that its supply did not trigger any significant effect on *VvHT1* and *VvHT5* expression in any of the studied cell lines. Mannitol, used as an osmotic control also failed to alter *VvHT1* and *VvHT5* expression. This excludes the transcriptional regulation of both transporter genes without hexose phosphorylation, and thereby the HXK-independent glucose signaling pathway (Figure 7 and Figure 8), confirming the role of HXK-dependent glucose- and mannose-signaling in all cell suspensions: WT, VvMSA-OE and VvMSA-RNAi (Figure 7 and Figure 8).

The unique and most remarkable difference between EC and NEC concerned the impact of 2DOG. This phosphorylatable, but not metabolizable, glucose analog repressed *VvHT1* and *VvHT5* to a little bit lesser extent than glucose or mannose in the three EC lines (WT, VvMSA-OE and VvMSA-RNAi) (Figure 7A,B and Figure 8A,B). Inversely, in NEC cells, 2DOG reduced *VvHT1* expression in WT and VvMSA-OE cells more strongly than glucose and mannose (Figure 7C,D). Most markedly, this glycolysis inhibitor did not affect *VvHT1* expression in *VvMSA-RNAi* non-embryogenic cells (Figure 7C,D). In addition, 2DOG induced *VvHT5* expression more than three-fold in the three NEC cell lines (WT, VvMSA-OE and VvMSA-RNAi) when compared to that of the same cells grown in control medium and under supply with other effectors (Figure 8C,D).

### 2.5. Analysis of Intracellular Metabolites under Sugar and Nitrate Depletion

**Figure 9:**
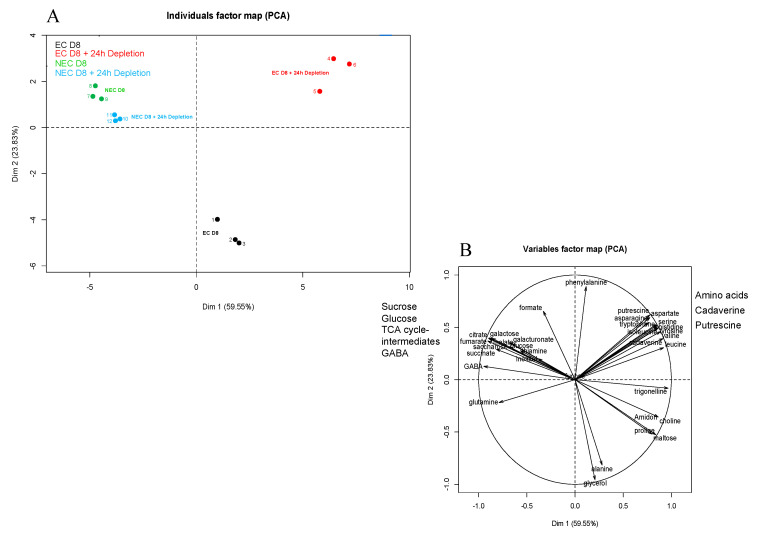
Principal component analysis (PCA) of measured cellular metabolites in embryogenic cells (EC) and non-embryogenic cells (NEC) grown under two conditions: 8 days of culture (D8) and 8 days of culture followed by 24 h of depletion (D8 + 24 h). (**A**) Individual map. Black-EC at D8; Red-EC at D8 plus 24 h depletion; Green-NEC at D8; Blue-NEC at D8 plus 24 h depletion (**B**) Correlation circles. The overall data of metabolite measurements were used, except glutamate, whose quantitation turned out to be impossible in the non-embryogenic cells. The soluble metabolites were quantified by ^1^H NMR, while starch was determined by an enzymatic method. Metabolite quantitation was performed on cells from three independent biological replicates.

The intracellular metabolite comparison of the EC and NEC was achieved by quantitative proton NMR analysis of cells harvested on the eighth day of subculture (D8) and at D8 + 24 h of culture on the respective minimal media. As already described by us [71], ^1^H NMR enabled the detection of 42 metabolites, from which 36 were identified. Principal component analysis (PCA) was used to unravel the differences between both cell types (Figure 9). The first PCA axis, corresponding to 59.55% of the variance, allowed the discrimination between NEC (D8 and D8 + 24 h of depletion) and EC (D8 and D8 + 24 h depletion). The second PCA axis responsible for 23.83% of the variance (Figure 9A), revealed the difference between EC at D8 and the other three groups, namely NEC at D8, NEC at D8 + 24 h depletion and EC at D8 + 24 h depletion.

**Figure 10:**
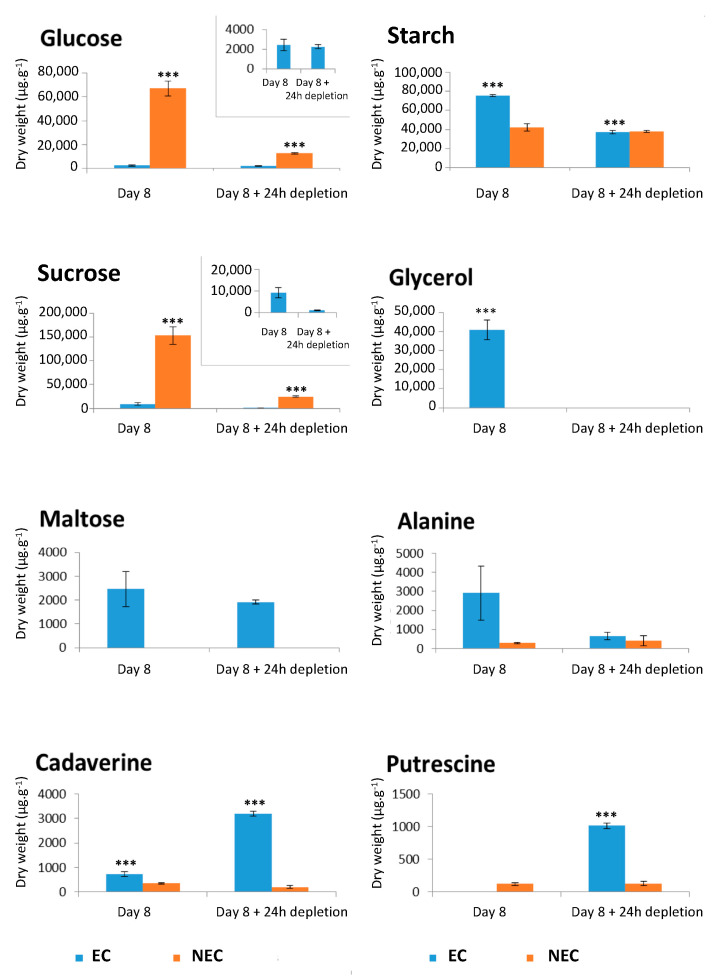
Shifts in concentration of soluble metabolites in embryogenic cells (EC) and non-embryogenic cells (NEC) grown under two conditions: 8 days of culture (D8) and 8 days of culture followed by 24 h of depletion (D8 + 24 h). The soluble metabolites were measured by ^1^H NMR. Starch was quantitated by an enzymatic method. The presented results correspond to the mean values of three independent biological replicates (±SEM). The asterisks denote the groups that were found significantly different by ANOVA with Tukey’s multiple comparison test (*** *p* < 0.001).

The EC at D8 were characterized by relatively high concentrations of glycerol and alanine, while those at D8 + 24 h depletion presented high concentrations of free amino acids and polyamines (cadaverine and putrescine) (Figure 10). At D8 + 24 h depletion, the NEC maintained higher concentrations of glucose and Krebs cycle intermediates (citrate, succinate, fumarate, malate, γ-aminobutyric acid, GABA), when compared to the EC under the same condition (Figure 9B and Figure S6). In EC, the 24 h depletion triggered a strong decrease of intracellular starch and sucrose concentrations, as well as moderate ones of succinate and malate. However, the concentrations of these metabolites were higher in NEC than in EC even after 24 h depletion: glucose > 6-fold; sucrose > 20-fold; succinate > 7-fold and malate > 20-fold (Figure 10 and Figure S6).

### 2.6. Maximal Activities of Glycolysis Enzymes under Different Treatments

In an attempt to determine the impact of VvMSA repression on reprogramming glucose metabolism, we established maximal activities of glycolysis enzymes in embryogenic and non-embryogenic, WT and VvMSA-RNAi cells. These activities were measured under the conditions of a pharmacological approach, which allowed us to register the most marked effects on *VvHT1* gene expression under “Depletion” (minimal medium), “Glucose” or “2DOG”-supply at D8 + 24 h deficiency for additional 6 h or 24 h of treatment, and compared to “Control” (complete medium) for the same time duration.

**Figure 11:**
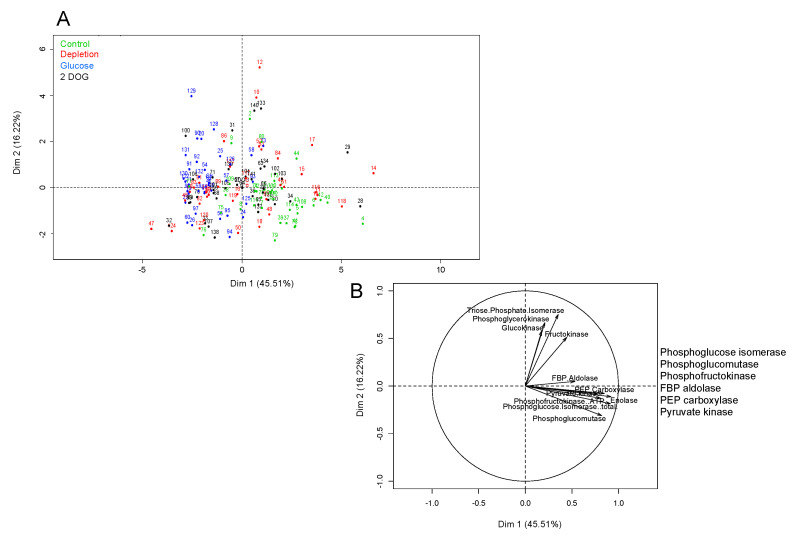
Principal component analysis (PCA) of measured enzyme activities in embryogenic WT and 35S::VvMSA-RNAi cells. (**A**) Individual map. (**B**) Correlation circles. The overall data of enzyme activity measurements were used. Studied conditions: Control-cells that were transfected in the complete GM^+^ culture medium for 30 h or 48 h. Depletion-cells that were transfected in the minimal GM° culture medium for 30 h or 48 h. Glucose-and 2DOG-cells that were transfected in the minimal GM° culture medium for 24 h and treated with 10 mM glucose or 0.9 mM 2DOG for additional time periods of 6 or 24 h. Green-control; red-depletion; blue-glucose; black-2DOG. Three independent biological repetitions were carried out for each of the studied conditions, with at least three technical replicates per biological repetition.

For embryogenic cells, the first axis of PCA with 45.51% variance allowed us to discriminate between the individual dots in “Control” and “Glucose” groups (Figure 11A), while those of “Depletion” and “2DOG” were scattered on the overall map. This suggests that the conditions “Depletion” and “2DOG” exerted little influence on the activity of the glycolysis enzymes in embryogenic cells. The first axis was correlated to the majority of the enzymes (PEPc; PK; ATP-PFK; PGI; PGM; FBP aldolase) (Figure 11B). It is therefore likely, that in a general manner, the cells of the “Control” group are characterized by stronger maximal activity of these enzymes than the cells of the “Glucose” group. The results of the ANOVA analysis imply that the VvMSA repression in embryogenic cells did not exert a significant effect on the activity of the different tested enzymes (Table S1). Inversely, there appeared to be a highly significant effect (*p* < 0.001), when the interaction between genotype and incubation time was extrapolated to the activity of FruK, and to a lesser extent to the activities of ATP-PFK, PGI and PGM (*p* < 0.05). This means that the activity of these enzymes evolved in a different manner after 6 h and 24 h of treatment of WT and VvMSA-RNAi cells (Figure 11B). Concerning FruK and PGM, their activities strongly decreased between 6 h and 24 h of treatment of wild-type cells, but not in VvMSA-RNAi cells in the “Depletion” condition. Likewise, the activities of PGI and PFK decreased between 6 h and 24 h in wild-type cells, but not in VvMSA-RNAi cells under the conditions “Depletion” and “2DOG”. The treatments produced a highly significant effect (*p* < 0.001) on the activities of ATP-PFK, PGM, PGI, enolase, and PEPc. Although at a less significant level, they also affected the activities of PK (*p* < 0.01) and FBP aldolase (*p* < 0.05) (Table S1). The activities of ATP-PFK, PGM, PGI and enolase were inhibited by glucose treatment (Figure 11B).

**Figure 12:**
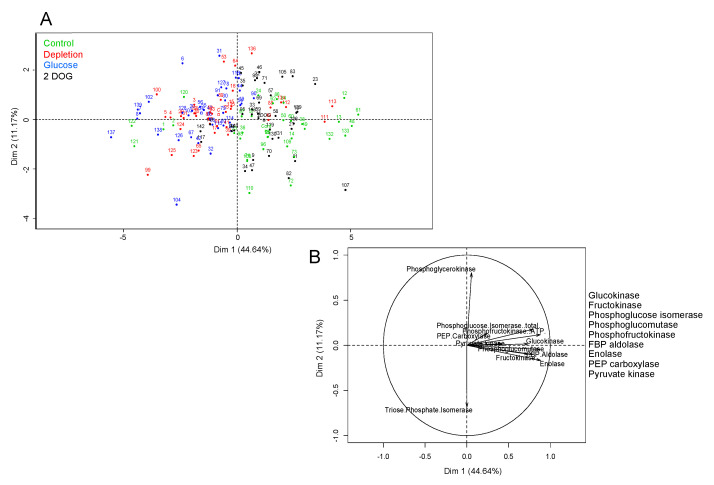
Principal component analysis (PCA) of measured enzyme activities in non-embryogenic WT and 35S::VvMSA-RNAi cells. (**A**) Individual map. (**B**) Correlation circles. The overall data of enzyme activity measurements were used. Studied conditions: Control-cells that were transfected in the complete GM^+^ culture medium for 30 h or 48 h. Depletion-cells that were transfected in the minimal GM° culture medium for 30 h or 48 h. Glucose and 2DOG-cells that were transfected in the minimal GM° culture medium for 24 h and treated with 10 mM glucose or 0.9 mM 2DOG for additional time periods of 6 or 24 h. Three independent biological repetitions were carried out for each of the studied conditions, with at least three technical replicates per biological repetition. Green-control; red-depletion; blue-glucose; black-2DOG.

For non-embryogenic cells, the first axis of PCA with 44.64% variance allowed us to discriminate between individual points corresponding to “Control” and “2DOG” on the one hand, and to “Depletion” and “Glucose” on the other (Figure 12). Even though separation was not totally clear-cut, this implied that the profile of the maximal enzyme activities of cells in the “Control” condition was closer to that of “2DOG” cells, while it displayed differences with the profiles of “Depletion” and “Glucose” groups. The first axis was positively correlated to the activities of almost all tested enzymes, except PGK and TPI. This means that most of the enzymes tended to display a higher level of maximal activity in “Control” and “2DOG” cells than in those of the “Depletion” and “Glucose” groups.

**Figure 13:**
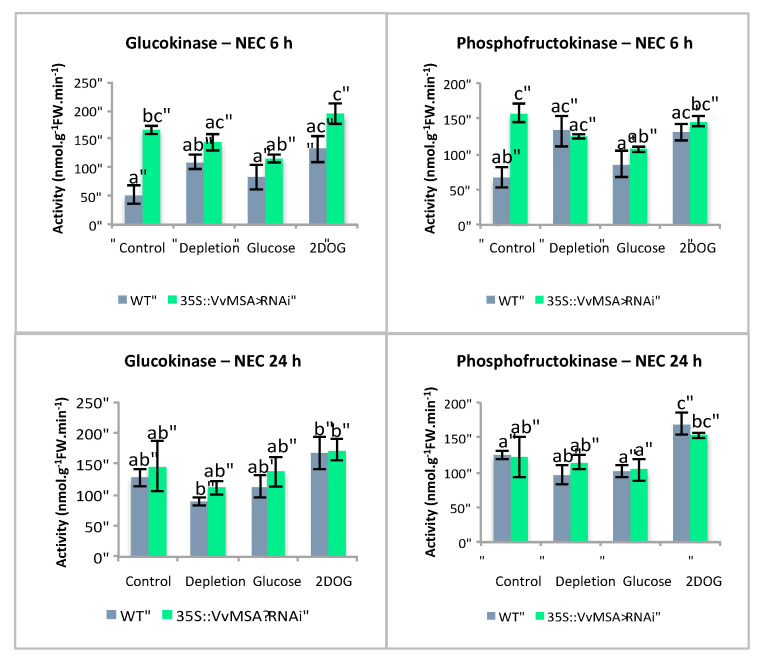
Activities of glycolysis-related enzymes in non-embryogenic (NEC) wild-type and 35S::VvMSA-RNAi cells. Studied conditions: Control—cells that were transfected in the complete GM^+^ culture medium or the Gb medium for 30 h or 48 h; Depletion—cells that were transfected in the minimal GM° culture medium for 30 h or 48 h; Glucose and 2DOG—cells that were transfected in the minimal GM° culture medium for 24 h and treated with 10 mM glucose or 0.9 mM 2DOG for additional time periods of 6 or 24 h. Three independent biological repetitions were carried out for each of the studied conditions, with at least three technical replicates per biological repetition. The letters denote the groups that were found significantly different by ANOVA and Tukey’s HSD test (*p* < 0.05).

VvMSA repression in non-embryogenic cells exerted a significant effect on the maximal activity of GluK and PGI (*p* < 0.001), PGK (*p* < 0.01), and PFK (*p* < 0.05) (Table S1). The activities of GluK, PFK (Figure 13) and PGI (Figure S7) were higher in VvMSA-RNAi cells than in wild-type cells of the “Control” group after 6 h of culture. In contrast, no significant difference in activity of the last three enzymes was found between WT and VvMSA-RNAi cells grown under the conditions “Depletion”, “Glucose” and “2DOG”, after 6 h and 24 h of culture. The treatments significantly impacted the overall enzyme activities (*p* < 0.001), except for PGK and TPI (Table S1). The most marked differences were found after 24 h of treatment. FruK, and to lesser extent GluK, activities were inhibited only under the condition “Depletion” (Figure 13). The activities of PGM, aldolase and enolase were inhibited under the conditions “Depletion” and “Glucose”, but not in those of “2DOG” and “Control”. In contrast, the activities of PFK and PGI were induced by 2DOG treatment, while they remained not significantly affected in the other three conditions. The activity of PK was inhibited by treatments with glucose and 2DOG, and was not affected by the two other conditions. The activity of PEPc appeared lightly inhibited under the conditions of “Depletion” and “Glucose”, but not under the two other conditions.

### 2.7. Glucose Absorption under Glucose and 2DOG Treatments

**Figure 14:**
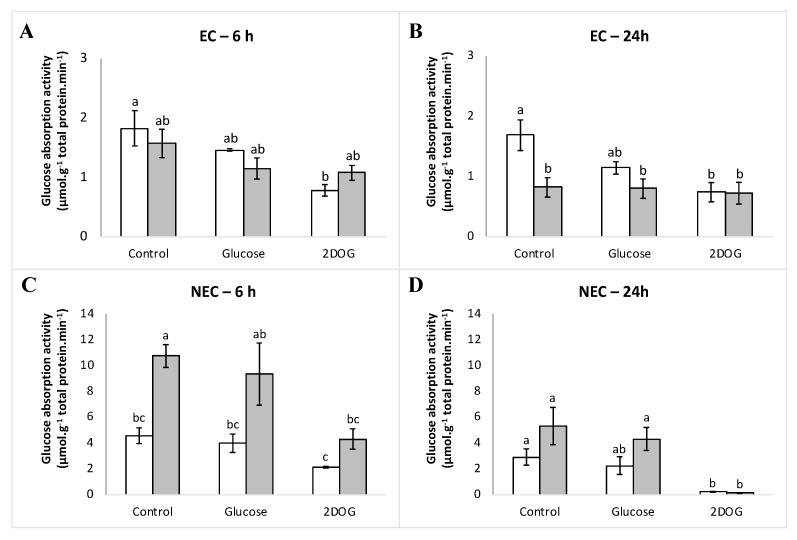
(**A**–**D**) Glucose absorption activity in embryogenic cells (EC) and non-embryogenic (NEC) WT and VvMSA-RNAi cells. □-WT; ■-VvMSA-RNAi. The embryogenic cells and the non-embryogenic cells were collected after 8 days of subculture and incubated for an additional 24 h in their respective minimal media, i.e., GM° minimal (EC) and Gb minimal (NEC). Sugar effectors were added afterwards and the treatment of EC was extended for 6 h, while that of NEC for 24 h. ^14^C-glucose uptake was measured at the 20th minute after the effector supply. Three independent biological repetitions were carried out for each of the studied conditions, with at least three technical replicates per biological repetition. The letters denote the groups that were found significantly different by ANOVA and Tukey’s HSD test (*p* < 0.05).

At the time point of 6 h, glucose absorption activity of embryogenic cells under control conditions was similar in both WT and VvMSA-RNAi cells (Figure 14A,B). It is likely that the treatment by glucose initiates light inhibition of absorption activity in both cell types at the time point of 6 h (20% and 31% respectively). The 2DOG treatment inhibited transport in WT cells (57% inhibition) more strongly than in VvMSA-RNAi cells, which remained the same as that under glucose treatment (31% inhibition). The inhibition propensity of glucose absorption in response to glucose administration appeared more marked after 24 h of treatment in wild-type cells (32% inhibition), while in VvMSA-RNAi cells the transport was already strongly reduced under control conditions (50%), and remained almost unchanged. Transport inhibition after 2DOG treatment of wild-type cells also remained practically unchanged between 6 h and 24 h time-points (57 vs. 56%, respectively). In *VvMSA-RNAi* cells, transport inhibition remained very close to that observed under control conditions (12% inhibition), which indicates that 2DOG has no effect on transport activity in those conditions.

For the glucose absorption activity of non-embryogenic cells under control conditions, we observed more transport (>two-fold) in *VvMSA-RNAi* cells compared to that of WT cells (Figure 14C,D). At the time point of 6 h, the glucose treatment triggered a weak inhibition of glucose absorption (decreased by 13 and 14%) in WT and VvMSA-RNAi cells, respectively). The treatment by 2DOG induced a more notable inhibition of the glucose transport (reduced by 54 and 60% in WT and VvMSA-RNAi cells, respectively). After the additional 24 h, the transport decreased in cells under control conditions, as well as in those treated either with glucose or 2DOG. It is noteworthy that the glucose transport in non-embryogenic VvMSA-RNAi cells was more strongly affected by extended depletion for 24 h (transport activity reduced by 50%) than in wild-type cells (reduction of 36% in transport activity). Transport inhibition under glucose treatment was slightly more marked after 24 h than that after 6 h (23% and 19% reduction of transport activity in WT and VvMSA-RNAi, respectively). In contrast, after 24 h, 2DOG triggered a nearly 100% inhibition of glucose absorption activity in both WT and VvMSA-RNAi cells.

In embryogenic cells, the part of passive transport represents ca. 25% and was very similar between the WT and VvMSA-RNAi cells analyzed at two time-points of 6 h and 24 h (Figure S8). Although passive transport also did not differ between non-embryogenic WT and VvMSA-RNAi cells, it was five-fold less than that in embryogenic cells—ca. 5%. The five-fold higher part of the passive transport in the embryogenic cells can probably be explained by their morphology and clumping propensity to form proembryonic aggregates. This hinders the washing efficacy and some portion of the radiolabelled glucose remains inside clumped cells.

### 2.8. Functional Complementation of the Arabidopsis Mutant abi8 with VvMSA

**Figure 15:**
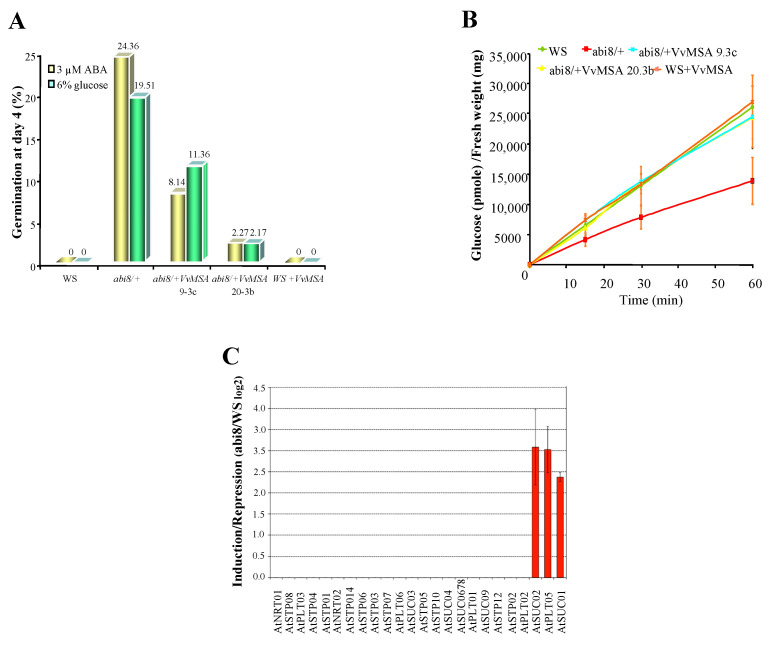
Functional complementation of the *Arabidopsis* mutant *abi8* by ectopic expression of *VvMSA*. (**A**) Sensitivity to ABA and glucose of wild ecotype WS, *abi8/+* mutant and *abi8*/+*VvMSA* 9.3c, *abi8*/+*VvMSA* 20.3b and WS + VvMSA transgenic lines homozygous for VvMSA was assayed by measuring germination after 4 days of incubation on minimal medium supplemented with 3 µM ABA and 6% glucose, respectively. Only the 25% *abi8* segregants show reduced sensitivity to ABA or glucose. (**B**) Glucose absorption activity of the same lines was measured on seedlings grown for 4 days on minimal medium to permit identification of *abi8* segregants based on their stunted growth and then transferred to minimal medium with 1% glucose for 4 days before harvesting. ^14^C-glucose uptake was measured on ten seedlings at four time points (0, 15, 30 and 60 min) and expressed as pmoles of glucose per mg of fresh weight. Three independent biological repetitions were carried out for each of the studied lines, with at least three technical replicates per biological repetition. (**C**) Expression analysis by macroarray of sugar transporters (monosaccharide, disaccharide, polyol) in Arabidopsis *abi8* mutant seedlings. The histograms show the mean of three independent experiments performed on 8-day-old seedlings (grown 4 days on minimal medium and an additional 4 days on minimal medium supplemented with 1% glucose). Expression values were initially normalized to those of three housekeeping genes of *Arabidopsis thaliana*, *AtGAPDH* (AT1G16300), *AtActin 1* (AT2G37620), *AtEF1β* (AT1G30230), and then reported to those of the wild genotype WS, in order to calculate the ratio of induction/repression.

As the ASR VvMSA interferes in the cross-talk of glucose and ABA signaling, the *Arabidopsis abi* mutants (insensitive to both glucose and ABA) appear as an appropriate model to study its role in these signal transduction pathways. The confirmed absence of an *ASR* gene in the *Arabidopsis thaliana* genome allowed us to apply “functional complementation” of *abi* mutants by overexpression of grape ASR, thereby avoiding the risk of post-transcriptional gene silencing by co-suppression. Two different *abi* mutants, affecting the APETALA2 transcription factor ABI4, and ABI8 encoding a protein of unknown function, were transformed with *p35S::VvMSA*. While *abi4* exists as a homozygous mutant, *abi8* is a seedling lethal and must be maintained as a heterozygote [72,73]. Several homozygous VvMSA-overexpressing lines, T3 and T4 generations, were obtained for both *abi4* and *abi8* mutants. Although transformed *abi4* + *VvMSA* enhanced its germination resistance to ABA and glucose (Figure S9), the overexpression of *VvMSA* partially restored the germination sensitivity of *abi8* to ABA and glucose (Figure 15A), reducing both germination and eventual cotyledon greening (Figure S10).

In order to unravel the mechanism of *abi8* rescue by VvMSA ectopic expression, the analysis of glucose absorption activity was performed in wild ecotype WS, *abi8*, *abi8* + VvMSA (line 9.3c and 20.3b), and revertant WS + VvMSA seedlings. Our results revealed an at least two-fold reduction of glucose transport activity in the mutant *abi8* in comparison with that of the wild ecotype WS. In addition, the glucose absorption was almost not affected in the *abi8* transgenic lines 9.3c and 20.3b overexpressing VvMSA (Figure 15B). Furthermore, the hybridization of our thematic macroarray specifically dedicated to grape sugar carriers [74] with WS and *abi8* probes, revealed such low expression of hexose transporters in the wild ecotype WS, that no inhibition of any of the studied *AtSTP* genes was detectable in the mutant *abi8* (Figure 15C). In contrast, a strong induction of gene expression of the sucrose transporters AtSUC1 and AtSUC2, as well as of the polyol transporter AtPLT5, was revealed in *abi8*.

## 3. Discussion

To decipher the mediator role of VvMSA in the pathways of glucose signaling through its target, the promoter of hexose transporter VvHT1, we successfully overexpressed and repressed this grape *ASR* in one embryogenic (EC) and one non-embryogenic (NEC) grapevine cell culture, thus creating a total of six cell lines: WT, VvMSA-OE and VvMSA-RNAi for each of them. Both EC and NEC have already been well characterized by us in terms of their cell fates, intracellular metabolites and enzyme activities of glycolysis [71]. The EC with organized cell proliferation coupled with predominant cytoplasmic growth, very low intracellular glucose concentration used with parsimony, and thereby low glycolysis flux, could represent a suitable model for high sensitivity to the glucose signal. Conversely, the NEC displaying anarchic cell proliferation coupled to cell expansion growth, supported both by important glycolysis flux and partial switch to fermentation, could be particularly sensitive to inhibitors of glucose metabolism.

The responsiveness of the two cellular models to glucose as a metabolic signal was studied throughout the expression of the grape ASR-target *VvHT1* gene, in parallel with another non-target hexose transporter gene *VvHT5* (Figure 8). It has been demonstrated by numerous pharmacological assays and global transcriptomic analyses that more than 2000 plant genes are regulated by glucose [75]. Keeping in mind that unlike phytohormones, sucrose, glucose and fructose are also basic substrates of different metabolic pathways, it appears difficult to discriminate between their function as signal molecules and their roles as the substrates/intermediates of plant metabolism [6,76]. The first clearly identified intracellular glucose sensor in plants is AtHXK1 of *Arabidopsis thaliana*, a cytosolic type B hexokinase associated with the external mitochondrion membrane [6,53,77,78,79]. The glucose sensor function of AtHXK1 has been confirmed by the characterization of the Arabidopsis *gin2* (glucose insensitive 2) mutant [14], and is involved in the regulation of many processes [80,81].

In order to gain a deeper insight into the VvMSA positioning in glucose signaling, we have set up a pharmacological approach by the administration of glucose and its analogs, which permits discrimination among the three well established pathways of glucose signal transduction. Glucose, mannose, 3OMG and the HXK competitive inhibitor mannoheptulose were administered at the same physiological concentration of 10 mM, while 2DOG was applied at 0.9 mM, in order to avoid any potential toxicity [82]. It is worth mentioning that the viability of the different cell lines under treatments with distinct effectors was demonstrated by Trypan blue staining, as described in Materials and Methods (data not shown). The real challenge lied in the set-up of a 24 h period of sugar and nitrate depletion, which started at the end of the exponential phase of cell proliferation (day 8). Such depletion compels the cells to consume their soluble intracellular sugars, thereby allowing them to remain under culture conditions that are both physiological and sufficient to observe the impact of glucose signaling on *VvHT1* expression.

The supply of glucose and mannoheptulose at equimolar concentrations of 10 mM in both EC and NEC cultures did not allow the inhibitor to override the repression of *VvHT1* strongly imposed by glucose (Figure S5). These results call into question the observed effects of 10 mM mannoheptulose versus the high non-physiological concentrations of 150 mM glucose and 150 mM sucrose [69,83], and the saturable nature of HXK.

The profiling of *VvHT1* and *VvHT5* expression regulation by sugar effectors in all studied WT and transgenic lines of both cell types allowed us to outline the glucose signaling pathways involved in the regulation of the gene expression of these two hexose transporters. It should be noted that in the studied grape-cell suspensions, compared to glucose, mannose consistently produced the same repression effect on *VvHT1* and more strongly inhibited the expression of *VvHT5* (Figure 7 and Figure 8). This appears to confirm mannose metabolic breakdown by cultured grape cells, a particular feature that has already been observed [83]. Taken together, the similar effects of glucose and mannose allowed us to provide evidence that *VvHT1* and *VvHT5* were both negatively regulated by the HXK-dependent pathway of glucose signaling, despite the missing effect of the HXK-specific inhibitor, mannoheptulose. This assumption was further assessed by the absence of any impact of mannitol as osmotic control. In this regard, 3OMG displaying a negligible phosphorylation by HXK has been supposed as not perceived by this glucose sensor, and thereby considered for analysis of the HXK-independent pathway [84]. In our study, 3OMG did not affect the gene expression of either hexose transporter, which implies that their regulation by sugar signaling necessitates at least the phosphorylation of glucose and excludes the HXK-independent glucose signaling pathway.

Our analysis of the glucose-signaling pathway, which is dependent only on HXK catalytic activity and downstream glycolysis intermediates/products, revealed strong differences in the sensitivity of both cell models to 2DOG. It has been shown in animal cells that 2DOG strongly inhibits isomerization of G6P and F6P [85]. Because of the low flux in the upper part of glycolysis in EC, it is likely that 2DOG exerts an extremely weak effect on their metabolism, contrary to that in NEC. In fact, this phosphorylatable, but not metabolizable, glucose analog repressed *VvHT1* and *VvHT5* similarly to glucose or mannose in the three EC lines. In non-embryogenic cells, the perturbation of glycolysis by 2DOG imposes the inhibition of *VvHT1* in WT and VvMSA-OE lines in the presence of grape ASR, while the repression of grape ASR in the VvMSA-RNAi cell line hinders the downregulation of *VvHT1*. These overall results provide a body of evidence for the regulation of *VvHT1* by glucose signaling via a glycolysis-dependent pathway. This pathway of glycolysis-dependent glucose signaling does not necessitate the sensor function, but only the catalytic function of HXK, and requires the mediation of grape ASR.

**Figure 16:**
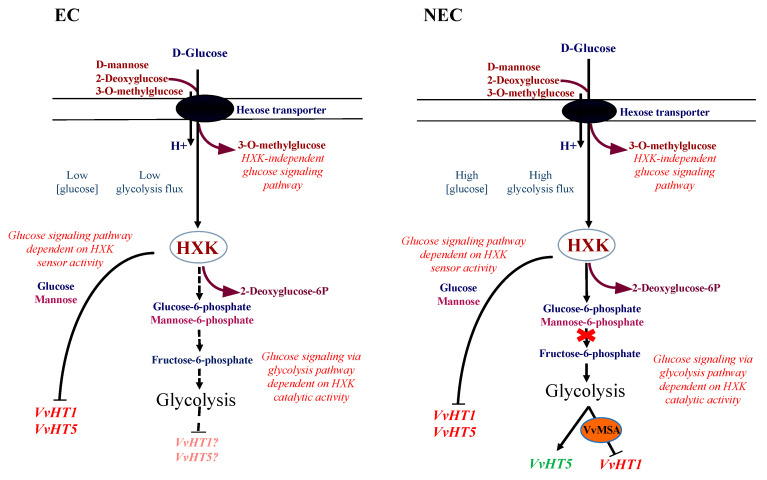
Hypotheses for the regulation of *VvHT1* and *VvHT5* expression in glucose signaling pathways. In embryogenic cells (EC), characterized by low glucose content and low glycolysis flux, the HXK-dependent glucose signaling triggers the inhibition of both the studied genes. Mannose may be detected by the cytosolic sensor HXK, and produces the same effects as those of glucose. In grape, mannose may also be phosphorylated as a substrate of HXK catalytic activity, and the resulting mannose-6-P could be metabolized, thereby allowing a glucose signaling pathway dependent on glycolysis. The less pronounced effect of 2DOG-blocked isomerization of glucose-6-phosphate to fructose-6-phosphate could be explained by its minimal impact on low glycolysis activity. In non-embryogenic cells (NEC), by presenting a high glucose content and a high glycolysis flux, inhibition by 2DOG of glucose-6-phosphate to fructose-6-phosphate isomerization leads to metabolic disruption. This metabolic switch results in *VvHT1* repression by glycolysis-dependent glucose signaling, which is mediated by its transcriptional regulator VvMSA. Inversely, the upregulation of the *VvHT5* expression by glycolysis-dependent glucose signaling appears independent from VvMSA.

Furthermore, 2DOG triggered the induction of *VvHT5* expression in all NEC lines, and this was in a grape-ASR-independent manner. It is worth noting that the gene expression of some pathogenesis-related proteins is upregulated by glucose signaling through the glycolysis-dependent pathway [86,87]. Other studies have reported *VvHT5* and its Arabidopsis homolog *AtSTP13* as induced in response to biotrophic and necrotrophic fungal infections [88,89,90]. In addition, the latter authors have shown that the molecular dialogue between Arabidopsis cells and *Botrytis cinerea* triggers major changes in host metabolism, suggesting enhanced glycolysis activity [91]. In this regard, our results provide evidence for the transcriptional regulation of *VvHT5* by glucose signaling via the glycolysis-dependent pathway, independently of grape ASR (Figure 16).

Metabolic analysis provided evidence that the sugar resources of EC at D8 were clearly more limited compared to those of NEC (Figure 10) [71]. During the depletion period, half of the starch content previously present in EC was consumed. Initially, very low contents of glucose and maltose were maintained nearly at the same level, allowing cells to assume moderate and synchronized cell proliferation. Inversely, alanine and glycerol were completely consumed. Alanine should be converted into pyruvate to provide carbon skeletons for the Krebs cycle. Glycerol was plausibly converted to DHAP (di-hydro acetone phosphate), which has to reintegrate into metabolism via either glycolysis or gluconeogenesis. Our overall results demonstrate that the profiles of intracellular metabolites of EC were more affected by the withdrawal of sugar and nitrates than those of NEC under deficiency [71].

Another important question is dealing with the issue of whether VvMSA repression could deeply affect the enzyme activities of glycolysis in response to the supplied sugars and sugar analogs. The study also gives support to our assumption of differential effects of the effectors between both cell types. Our analysis implies that the maximal activities of two enzymes, GluK and PFK, are significantly more increased in *VvMSA-RNAi* cells than in wild-type cells after 30 h of depletion (i.e., when comparing the effect of the transfer in a minimal medium to that of the control complete medium). The difference fades away 48 h after their transfer. Enzyme activities in embryogenic cells are mostly affected by glucose treatment (inhibition), but not by 2DOG. This shows that in cells which are adapted to low glucose levels and display weak flux in the upper part of the pathway, the addition of 10 mM glucose induces an important modification in the cell metabolism—the reprogramming of glycolysis enzymes. In non-embryogenic cells the activities of glycolysis enzymes are relatively similar under the conditions of “depletion” and “glucose”, but still significantly different from those under 2DOG treatment. This demonstrates that the treatment by 10 mM glucose is not likely to significantly influence the metabolism of these cells, which are habituated to high glucose concentrations. Conversely, the treatment by 2DOG induces the reprogramming of glycolytic enzymes, and probably reflects flux perturbation in glycolysis. This result appears to corroborate the assumption that VvMSA repression perturbs adaptive cell metabolism in response to sugar and nitrate starvation.

As glucose is not only a metabolic signal, but also the specific substrate of the VvHT1 transporter (target of the grape ASR), the analysis of its absorption activity in all cell lines of both EC and NEC types completed our study. In the latter context, as previously reported in yeast and grape cell cultures, VvHT1 operates as a high-affinity (Km = 50 mM Glc) and broad-specificity monosaccharide-proton cotransport system [69,92]. These VvHT1 features have been further corroborated by high-affinity absorption corresponding to similar Km values determined for hexose uptake in cell suspensions of tobacco [93], carrot [94], olive [59], in guard cell protoplasts of pea [95], as well as in yeast, lower and higher plants expressing monosaccharide transporters (MSTs) [58]. This high affinity measured for VvHT1 as an H^+^-dependent monosaccharide transporter may be important for cell growth in media where the sugar supply rapidly becomes limiting [69]. The latter assumption implies that the glucose transporter VvHT1 should be responsive to physiological concentrations of glucose (10 mM) at both levels, gene expression and protein activity, as already reported [77].

It is likely that the glucose supply at low physiological concentration (10 mM), which strongly inhibited *VvHT1* and *VvHT5*, does not affect glucose transport in a significant manner. The transport was mainly affected by 2DOG treatment (0.9 mM), which implies that the latter is preferentially regulated in response to modifications in the cell energy metabolism. The comparison of the effects of glucose signaling on *VvHT1* and *VvHT5* expression and the impacts of this same signal on the global glucose uptake strongly suggest a decoupling in the regulation of gene expression and absorption activity of these hexose transporters. Our results are in accordance with the already reported lack of correspondence between transcript and protein steady-state levels of the VvHT1 transporter [69]. This assumption could be partially assessed by the relatively long half-life of these proteins compared to that of their transcripts. The authors of the latter have also demonstrated by immunoblot analysis the post-transcriptional repression of the VvHT1 protein in terms of reduced transporter presence on the plasma membrane at high 150 mM glucose concentration. Furthermore, it has been suggested that high glucose levels may repress glucose transport activity at the protein level, triggering inactivation, mistargeting, and/or proteolysis of VvHT1 [69]. Obviously, this phenomenon coping with glucose concentration as substrate, but not as metabolic signal, is common for rate-limiting proteins, such as transporters, and has been well demonstrated in yeast, where it is denoted as carbon inactivation [96,97,98].

The isolation of glucose-insensitive or glucose-hypersensitive mutants and the characterization of genes that are affected by these mutations has highlighted close relationships between glucose-signaling and ABA-dependent and ethylene-dependent signaling in the control of the early phases of plant development [99,100,101,102,103,104,105]. Several loci have been associated with both sugar- and ABA-responses, which implies a common transduction pathway of sugar and ABA signals. These mutants have been classified into two groups: the first ones are affected in key ABA biosynthetic enzymes (*ABA1*, *ABA2/GIN1/ISI1/SIS4*, and *ABA3/GIN5/LOS5*), and the second ones are affected in the sensitivity to ABA and act as transcription factors (*ABI3*, *ABI4/GIN6/ISI3/SIS5/SUN6*, *ABI5*) [103,106,107]. Because *ABA3* is an ABA biosynthetic gene, it is likely that accumulation of ABA is a prerequisite for triggering a response to a perceived “glucose” signal [100]. The latter assumption has been corroborated by the finding of other glucose-insensitive *aba* mutants—*aba1-1*, *aba2-1* and *aba3-2* [100]. Moreover, the supplementation of ABA at a physiological concentration (100 nM) in the culture medium overrides the glucose insensitivity of the mutants *gin5/aba3* [103]. Interestingly, the three ABI genes encoding different transcription factors (*ABI3*, *ABI4 and ABI5)* appear to function in the same signaling pathway for mediating seed germination and early seedling development in response to ABA [108]. In contrast, *ABI8/ELD1/KOB1* appears to function downstream of *EIN2* and *EIN3* in ethylene-dependent signaling, and possibly in a separate pathway from those requiring action of ABI transcription factors to regulate ABA effects on germination [73].

The overexpression of tomato *ASR*, *SlASR1,* in Arabidopsis has been demonstrated to decrease germination sensitivity to ABA and glucose, and thereby to confer an *abi* mutant phenotype [109]. Through using chromatin immunoprecipitation assay with SlASR1 antibody, the same authors have reported that tomato ASR1 binds to the ABI4 promoter in vivo and specifically recognizes the CE1 element. The latter appears to be not only the binding sequence of ABI4 target genes, but also a *cis*-element of the proper promoter of *ABI4*, which is responsible for the positive feedback of its transcription autoregulation. The *ABI4* gene encodes an APETALA 2 (AP2) domain transcription factor, which is involved in the fine-tuning of seed development and germination [72,110,111]. However, the ChIP approach has not revealed any SlASR1 binding activity to promoter sequences of genes that are directly regulated by ABI3 and ABI5 transcription factors. The proposed model suggests that tomato ASR1 may compete with ABI4 for the same binding sequence. Concerning our attempt for functional complementation of *abi4* mutant, the ectopic expression of grape ASR failed to restore seed germination sensitivity to ABA and glucose. Despite the difference between the ABI4 binding sequence CE1 (CACCG), partially overlapping that of SlASR1 (C_2-3_(C/G)A, and that identified by us for VvMSA, as a combination of “Sucrose box 3” (AAATCA) and SURE1 (ATAGAAA) elements in the native context of *VvHT1* promoter [32], both ASRs affect germination resistance phenotypes. A plausible explanation may be the high DNA-binding ability of ASR proteins, and their capacity to form protein heterodimers. VvMSA, an architectural type transcription factor, might affect the recruitment of ABI4 required for the induction of a subset of genes.

It is worth noting that VvMSA provides partial functional complementation of the Arabidopsis *abi8* mutant. *ABI8* appears to be regulated in an ABA- and ethylene-dependent signaling pathway, different from the strictly ABA-dependent one, which requires *ABI3*, *ABI4* and *ABI5* genes [73]. We provide evidence for a plausible molecular mechanism relying on a two-fold reduced glucose uptake by *abi8* mutants compared to that of wild ecotype WS- and *VvMSA*-overexpressing lines. These results raise the question of whether glucose uptake in the *abi8* mutant is sufficient to yield an intracellular signal. They are further corroborated by the very low gene expression of hexose transporters versus the strong upregulation of two sucrose carriers and one polyol transporter in the mutant. In fact, the mutants require glucose, but not sucrose to maintain root growth. Moreover, our previous studies have shown that *abi8* growth is not only dependent on low concentrations of glucose, but is also resistant to the inhibitory effects of high glucose, suggesting a defect in sugar signaling and/or transport. We have already demonstrated that the expression of vacuolar and cytosolic invertases, as well as of another cytosolic sucrose-cleaving enzyme, sucrose synthase, was reduced in mutant roots [73]. Our unpublished results imply that ABI8 is involved in the Golgi trafficking of membrane proteins and thus may exert an indirect effect on glucose and ABA response. It is possible that they upregulate sucrose transporters as a response to being “starved” for sugars because they are inefficient at inserting the transporters into membranes. The presented results provide evidence for reduced glucose uptake of *abi8*, which combined with inefficient intracellular sucrose cleavage, caused its stunted root phenotype.

## 4. Materials and Methods

### 4.1. Embryogenic and Non-Embryogenic Cell Cultures

The grapevine embryogenic cells (EC) originate from the rootstock hybrid 41B, the most commonly used rootstock in the vineyards of Champagne (a hybrid between *Vitis vinifera* L. cv. Chasselas × *Vitis berlandieri* P.), and were used for genetic transformation via somatic embryogenesis and the subsequent regeneration of transgenic vitroplants, from which were derived the non-embryogenic cells (NEC). EC and NEC were cultured into their specific media permitting the maintenance of growth and metabolic specificity of each cell type, as previously described by us [71]. The 41B embryogenic cells were transformed with the 35S::VvMSA-RNAi construct via *Agrobacterium tumefaciens* strain EHA 105, coculture of the grape 41B cells with the bacteria, for 60 h on the above-mentioned solid medium, and selection of transformed cells on paromomycin (2 µg·mL^−1^) [112]. The somatic embryogenesis of 41B cells was induced by their subculture into the same fresh medium depleted of auxin and at 26 °C. The pretreatment of EC before the supply of effectors was carried out after 8 days of subculture by washing the cells three times in a minimal GM medium (i.e., 0.5× Murashige Skoog). The whole volume of the medium was aspirated without disturbing the cells that were immediately overlaid with an equivalent volume of the same medium. The cell suspension was then placed on an orbital shaker for 15 min at 110 rpm. After that, 0.2 mL of packed cell volume (PCV) was resuspended in 3 mL of minimal GM medium in a 6-well cell culture plate (Nunc). The pretreatment of non-embryogenic cells was carried out under the same procedure as that of the EC after 8 days of subculture, but with a minimal Gamborg medium. As a next step, 0.3 mL of the PCV cells were resuspended in 3 mL of minimal Gamborg medium in a 6-well cell culture plate (Nunc). After pretreatment, both embryogenic and non-embryogenic cells were incubated for 24 h under agitation at 110 rpm, in the dark, and at a constant temperature of 21 °C.

### 4.2. Treatment with Glucose and Glucose Analogs of Embryogenic and Non-Embryogenic Cells

The effectors were supplied by addition of 30 µL of 1M solutions of glucose, mannose, 3-O-methylglucose (3OMG) or mannitol, and of 27 µL of 100 mM 2-déoxyglucose (2DOG) per microplate well containing 3 mL of cell suspension (10 mM final concentration of glucose, mannose, 3OMG, mannitol, and 0.9 mM final concentration of 2DOG). The cells were incubated for an additional 24 h under agitation at 110 rpm, in the dark at a constant temperature of 21 °C. Our experimental protocol consisted of maintaining cells in a minimal medium devoid of sugars and nitrates for 24 h, just before initiating treatments with effectors for an additional 6 h and 24 h (for a total of 30 h or 48 h). In order to evaluate the impact of the transfer of cells in a minimal medium (Depletion), we comparatively analyzed enzyme activities of cells in complete medium (Control). The experimental conditions were denoted so far as “Control” (transfected cells in complete culture medium during 24 h plus an additional 6 h or 24 h), “Depletion” (transfected cells in minimal culture medium during 24 h plus an additional 6 h or 24 h), “Glucose” and “2DOG” (transfected cells in minimal culture medium during 24 h that were treated with 10 mM glucose or 0.9 M 2DOG for an additional 6 h or 24 h, respectively). The cells were collected under partial vacuum on glass microfiber filters (Fisherbrand) mounted in a filtration unit (Millipore) and then rinsed with 10 mL of the respective minimal medium for each of the two cell types. Before sample collection, cell viability was estimated by Trypan blue which stains dead cells only. One drop of cell suspension was directly mixed with equal volume of 0.4% Trypan blue solution in ddH_2_O in a Malassez cell-counting chamber and observed by microscope (Olympus BH; 100×).

### 4.3. Germination Assays

Germination assays were performed with seeds that were surface sterilized in 5% hypochlorite and 0.02% Triton X-100 and then rinsed several times with sterile water before plating on minimal medium containing 0.7% (*w*/*v*) agar supplemented with different concentrations of ABA or glucose [73]. The dishes were incubated for 3 days at 4 °C to break any residual dormancy and then transferred to 22 °C in continuous light (50 to 70 µE·m^−2^·s^−1^). Germination was scored as emergence of entire seedling from seed coat after 4 days. Transformation of WT, *abi4-1* (AT2G40220), and *abi8/+* (AT3G08550) mutants with 35S::VvMSA was performed using *Agrobacterium tumefaciens* by floral dip in planta transformation [113]. Independent transformants were selected on 40 µg/mL kanamycin-supplemented minimal medium in order to identify homozygous lines. Germination analyses were performed on 150–200 seeds per T3 homozygous line.

### 4.4. Glucose Uptake into Embryogenic and Non-Embryogenic Cells, and Arabidopsis Seedlings

For each of the studied conditions, 225 mg of EC or 450 mg of NEC, that had previously been filtered and rinsed, was resuspended in 12 mL of minimal GM medium or minimal Gb medium, respectively. The cells were incubated in equilibration buffer containing 0.2 M glucose for 30 min at 115 rpm orbital agitation. After equilibration, ^14^C glucose was added at 0.1 μCi·mL^−1^ specific radioactivity and aliquots were taken in triplicate every ten minutes, from 0 min to 30 min. The cells were collected by filtration onto glass microfiber filters (Fisherbrand, Houston, TX, USA) and washed three times with the respective equilibration medium. In parallel, the involvement of the proton-motive force in sugar uptake was evaluated by addition of 20 μM carbonyl cyanide m-chlorophenylhydrazone (CCCP) to the incubation buffer 10 min before the input of radiolabelled glucose.

Glucose absorption activity of the *Arabidopsis* wild ecotype WS (Wasilewskaja), *abi8* mutant and their transgenic lines homozygous for grape ASR, VvMSA, was measured on seedlings grown for 4 days on minimal medium to permit identification of *abi8* segregants based on their stunted growth and then transferred to minimal medium with 1% glucose for an additional 4 days before harvesting. ^14^C-glucose uptake was measured on ten seedlings, previously weighed to determine the exact fresh weight, and incubated after gentle infiltration into 200 µL of Gamborg B5 medium, for 30 min, at room temperature. In order to trigger absorption, the used incubation medium was replaced by 200 µL of a fresh one containing 50 µM glucose and 0.1 µCi ^14^C-glucose. Radioactivity was measured at four time points (0, 15, 30 and 60 min), after aspiration of reaction medium and five washes with cold Gamborg B5 medium without sugar. Absorption activity was expressed as pmoles of glucose per mg of fresh weight. At least three independent biological repetitions were carried out for each of the studied lines, with three technical replicates per biological repetition.

The samples were incubated in digestion buffer (13% perchloric acid (*v*/*v*), 12% hydrogen peroxide (*v*/*v*), 0.04% Triton X-100 (*v*/*v*)) for 24 h at 60 °C. Incorporated radioactivity was determined by liquid scintillation counting (Tri-Carb 2910 TR, PerkinElmer, Waltham, MA, USA).

### 4.5. Real-Time RT-qPCR Analysis

Reverse transcription was carried out on 2 µg of DNase-treated total RNA according to manufacturer protocol (Promega, Madison, WI, USA). RTqPCR was performed as described previously [15,71]. Accession numbers and primers of studied genes *VvMSA* (Q94G23_VITVI) F-GCATGTGTGCTTGTTGTGTAA and R-TCACAAGGACACACAGAGAGA; *VvHT1* (E3VWT5_VITVI) F-TATATTGGTGTCGGATTGCT and R-AAGAAGAACATAGGGAAAGC; *VvHT5* (Q3L7K6_VITVI) F-TAGTGATGCGTCCCTCTACTCA and R-CTTCCAGCAAGAGCAATCGAC, and the reference genes *VvActin* (Q94KC1_VITVI) F-GCATCCCTCAGCACCTTCCA and R-AACCCCACCTCAACACATCTCC; *VvGAPDH* (F6GSG7_VITVI) F-TTCTCGTTGAGGGCTATTCCA and R-CCACAGACTTCATCGGTGACA.

### 4.6. Macroarray Analysis

Total RNA isolation, cloning of specific cDNA fragments for genes of interest, reference genes, macroarray spotting and hybridization were performed as described previously [74]. In the latter publication, we provided the accession numbers and the primer sequences of all sugar transporter genes. After quantification (Typhoon TRIO Imager, GE Healthcare, UK), signals for each gene of interest were normalized using the mean of the four reference genes. Their accession numbers and primer sequences were: *VvGAPDH* (F6GSG7_VITVI) F-CGACCATTGTTACTGCTGT and R-GAAATCCAGGGGCAAAAC; *VvActin* (Q94KC1_VITVI) F-AGCTGGAAACTGCAAAGAGCAG and R-ACAACGGAATCTCTCAGCTCCA; *VvEF1a* (A5AFS1_VITVI) F-GAACTGGGTGCTTGATAGGC and R-AACCAAAATATCCGGAGTAAAAGA; *VvEF1γ* (A0A438CSH7_VITVI) F-AGCTTTTACCGCGGGCAAGAGATACC and R-TTTGGATAGGTAACGTATCACTTAAATAAC.

### 4.7. Metabolomic Analysis

For the ^1^H-NMR analysis, polar metabolites were extracted, titrated, lyophilized (EZ Dry-FTS system), solubilized and pretreated as described by [114]. ^1^H-NMR spectra were recorded at 500.16 MHz and 300 K on a Bruker Avance III spectrometer using a 5 mm inverse probe and an electronic reference for quantification (Digital ERETIC, Bruker TopSpin 3.0). The assignments of metabolites in the NMR spectra were made by comparing the proton chemical shifts with literature values [115,116,117], with the spectra of authentic compounds recorded under the same buffer conditions and by spiking the samples with standards. Metabolite concentrations in the NMR tube were calculated using Analytical Profiler mode of AMIX software (version 3.9.10, Bruker, Billerica, MA, USA) for calculation of resonance areas, followed by data export to Excel software. The 2D-homonuclear correlation spectroscopy (^1^H-^1^H COSY) experiments were carried out to verify the identity of known compounds and to check whether unknown signals really correspond to different compounds. Starch was determined as described previously [118].

### 4.8. Enzyme Activities

Extraction was performed as described previously [119]. All enzyme activities were carried out on a robotized platform [120], and assayed as described respectively: phosphoglucose isomerase—PGI [121]; enolase and triose phosphate isomerase—TPI [122]; phosphoglucomutase—PGM [123]; pyruvate kinase—PK, glucokinase—GK, fructokinase—FK, phosphoenolpyruvate carboxylase—PEPC and ATP-dependent phosphofructokinase—PFK [120]; phosphoglycerokinase—PGK [124]; aldolase [125]. Proteins were quantified by the method of Bearden [126].

## 5. Conclusions

We deciphered the mediator role of the grape ASR protein, VvMSA, in the pathways of glucose signaling through the regulation of its target, the promoter of hexose transporter VvHT1. By using different glucose analogs to discriminate between distinct pathways of glucose signal transduction, we revealed VvMSA positioning as a transcriptional regulator of the glucose transporter gene *VvHT1* in a glycolysis-dependent glucose signaling pathway. The analysis of the effects of *VvMSA* overexpression/repression on glucose transport and the activities of glycolysis enzymes revealed its role as a mediator in the interplay of glucose metabolism, transport and signaling. Ectopic expression of *VvMSA* in the *Arabidopsis* mutant *abi8* resulted in its partial functional complementation by improving glucose absorption activity, thereby suggesting common roles of these two proteins in the repression of seed germination through the regulation of metabolic trafficking. We revealed that, in grape cells under heterotrophic culture conditions, the repression of the major hexose transporter *VvHT1* via the glycolysis-dependent glucose signaling requires VvMSA, while the expression inhibition of another hexose transporter *VvHT5* is independent of VvMSA. The overall results permitted us to build a model for the differential regulation of gene expression of the hexose transporters VvHT1 and VvHT5 in HXK-dependent glucose signaling pathways.

## Data Availability

Not applicable.

## Supplementary Materials

The following supporting information can be downloaded at: https://www.mdpi.com/article/10.3390/ijms23116194/s1.
