# Supplementary material for: Grape ASR Regulates Glucose Transport, Metabolism and Signaling
Source: Int J Mol Sci. 2022 May 31;23(11):6194. doi: 10.3390/ijms23116194 (PMC9181829; doi:10.3390/ijms23116194)
Supplement: Supplementary file 1 [file ijms-23-06194-s001.zip › ijms-1737505-supplementary.pdf]

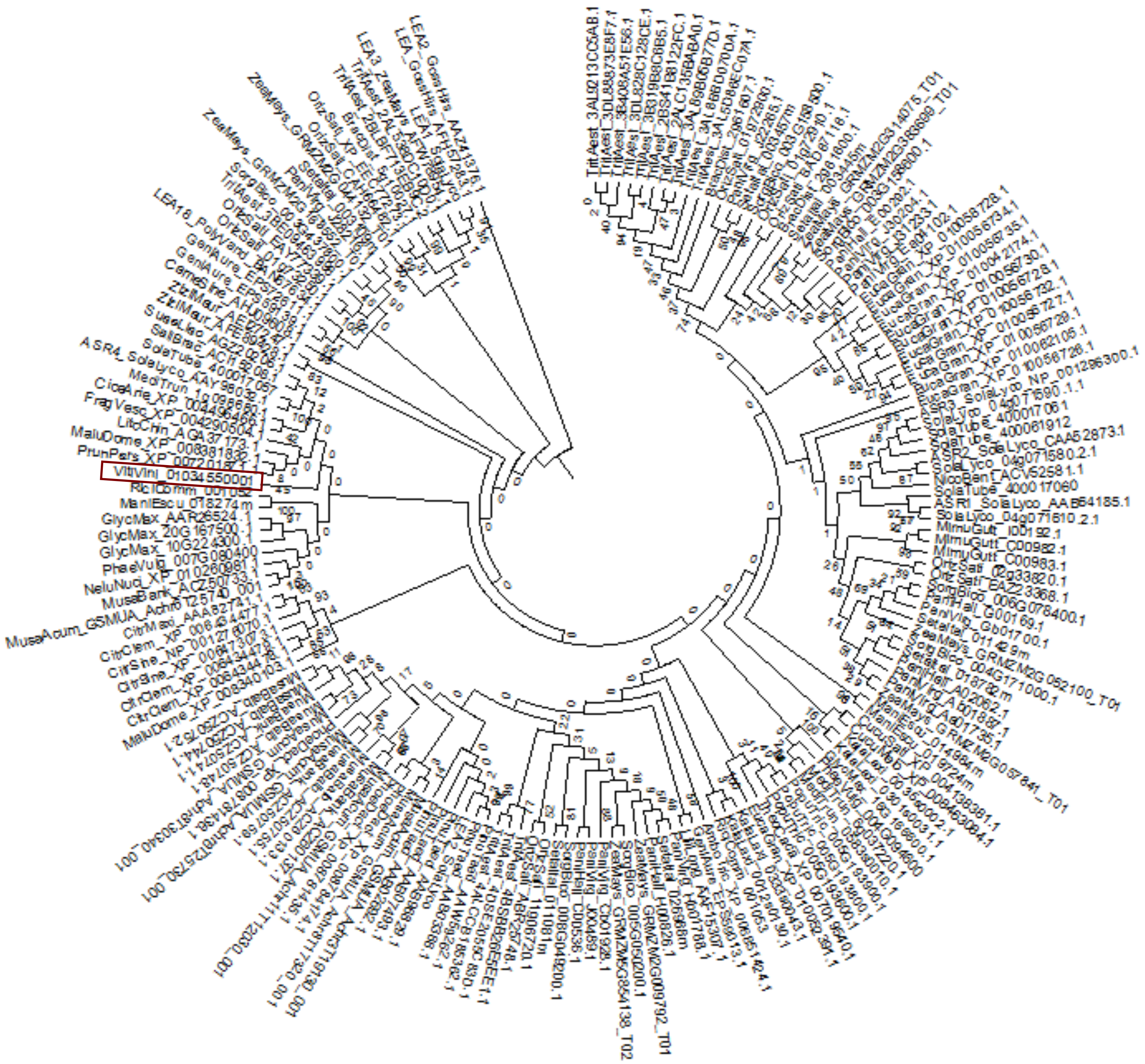

Figure S1. Phylogenetic tree of ASRs.

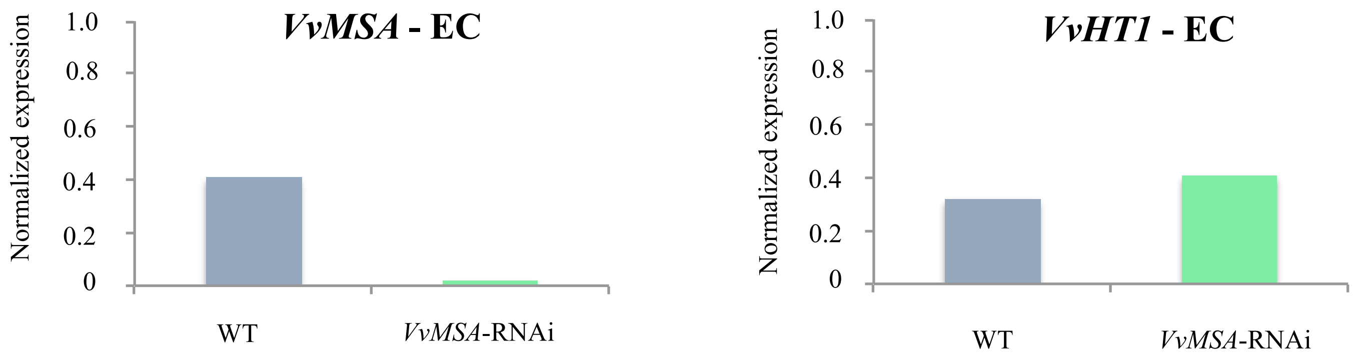

Figure S2. Long-term expression of *VvMSA* and *VvHT1* in embryogenic cells (EC) at day 8 after subculture. The expression of both genes was normalized to that of the housekeeping gene *VvActin*.

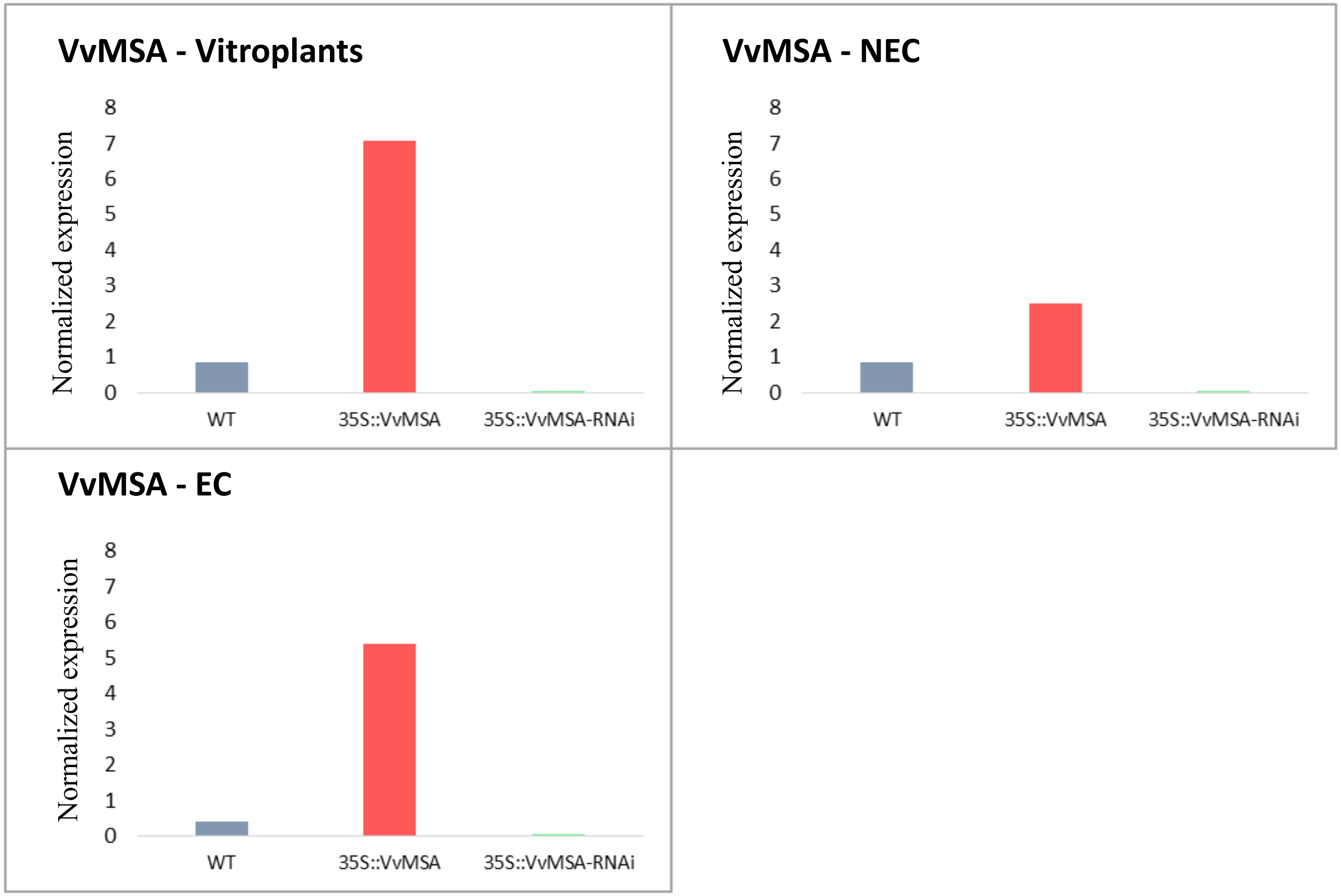

Figure S3. Expression of *VvMSA* in virtroplant leaves, embryogenic and non-embryogenic 41B WT, *35S::VvMSA* and *35S::VvMSA*-RNAi. The expression of VvMSA in vitroplant leaves was analyzed after their micropropagataion, and that in embryogenic and non embryogenic cells after 8 days of subculture. *VvMSA* expression normalized to that of the housekeeping gene *VvActin.*

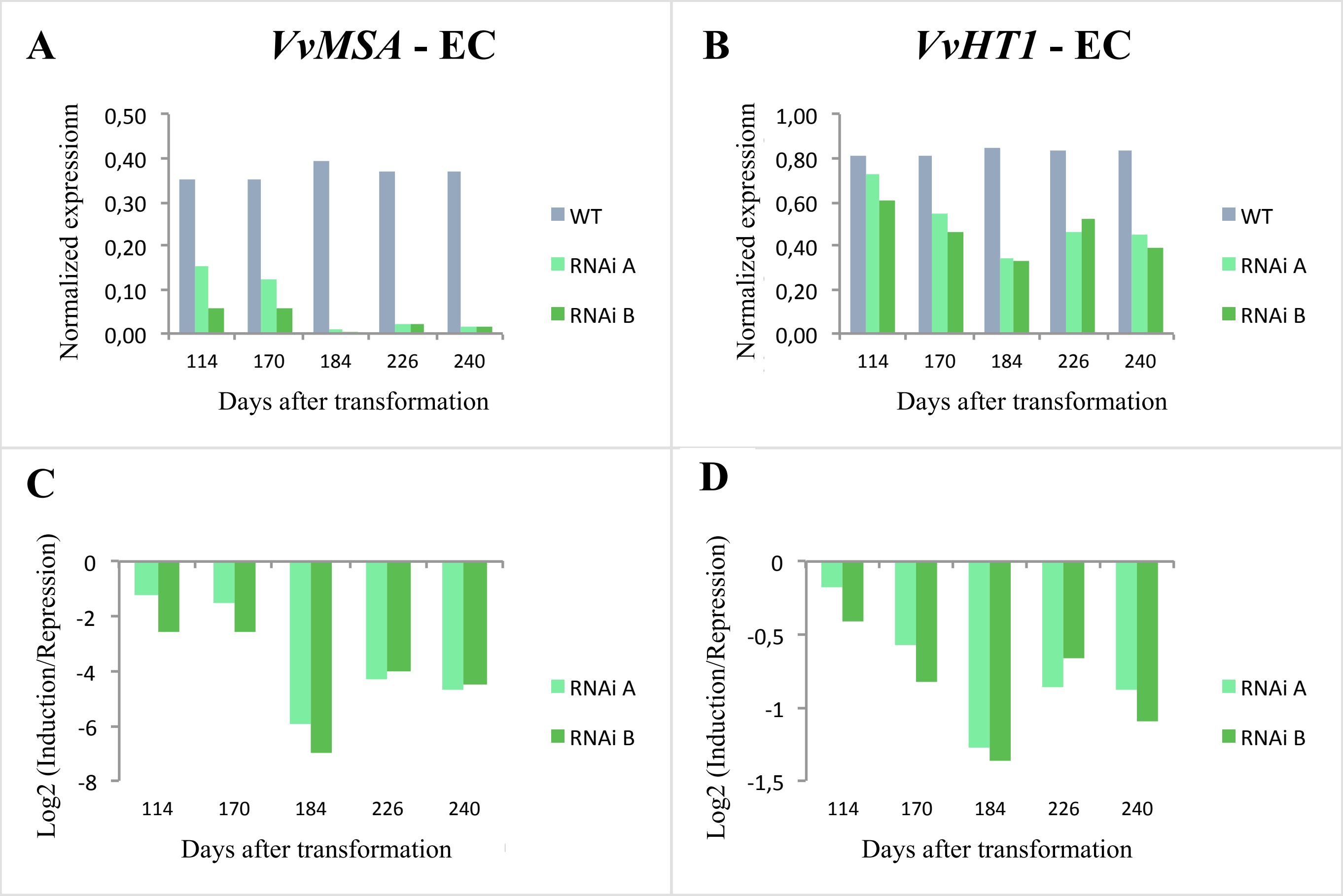

Figure S4. Evolution of *VvMSA* and *VvHT1* expression in embryogenic cells of wild-type and *VvMSA*-RNAi from two new independent genetic transformations, RNAi A and RNAi B.

The expression of both genes is normalized to that of the housekeeping gene *VvActin* and reported to the wild-type control condition used to calculate the ratio of induction/repression. Normalized expression (**A** and **B**) and induction/repression (**C** and **D**) calculated by the methods of ΔCt and 2-ΔΔCt respectively.

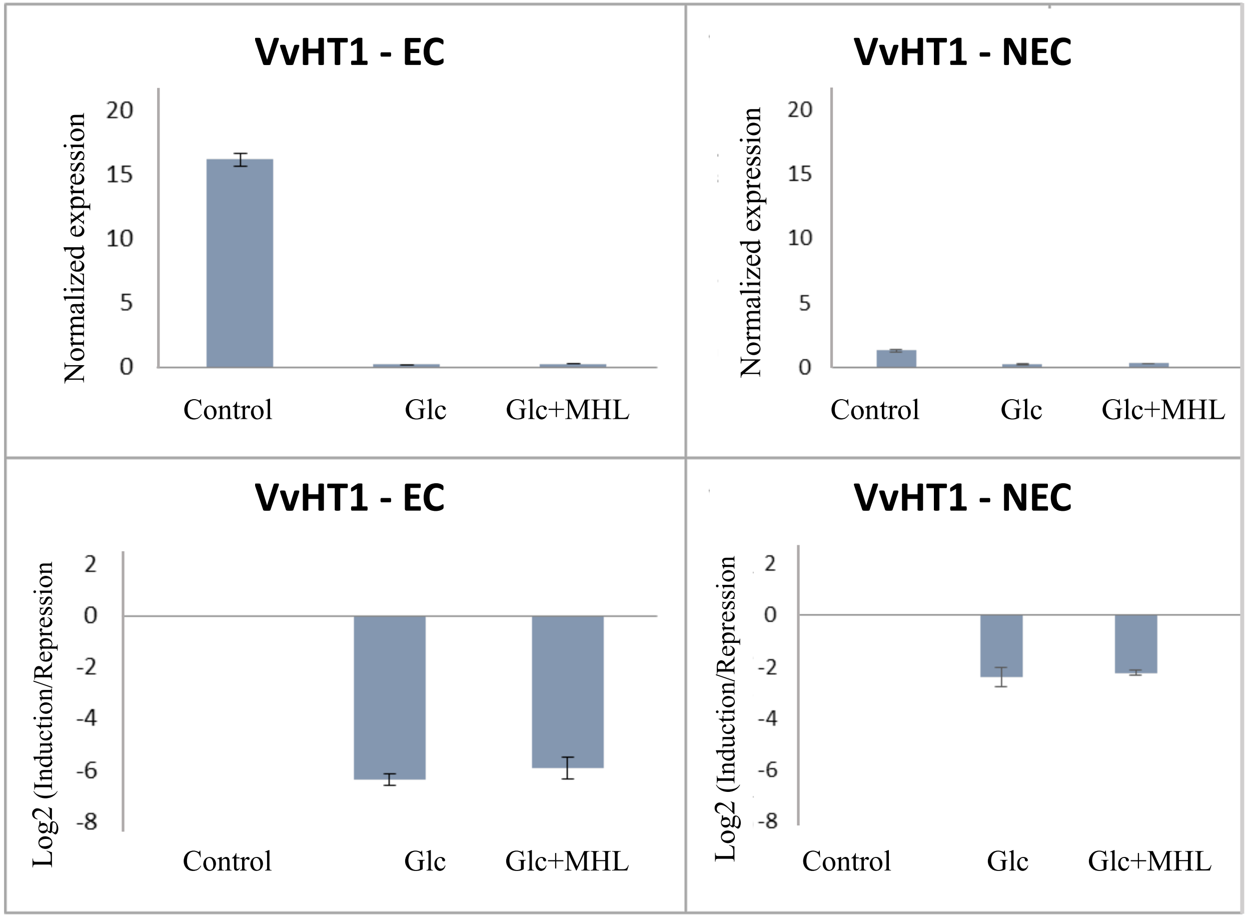

Figure S5. Expression of *VvHT1* in embryogenic (EC) and non-embryogenic (NEC) cells in response to different treatments. Glucose (Glc) and mannoheptulose (MHL) concentration – 10 mM. The cells were collected after 8 days of subculture and incubated for additional 24 hours in minimal Gb medium. Sugar effectors were then added and the treatement was extended to 6 hours for EC and 24 hours for NEC. The gene expression of *VvHT1* is normalized to that of the housekeeping gene *VvActin* and reported to the control condition used to calculate the ratio of induction/repression. Normalized *VvHT1* expression to *VvActi*n and induction/repression were calculated by the methods of ΔCt and 2-ΔΔCt respectively. The presented results correspond to the mean values of two independent biological experiments (±SEM).

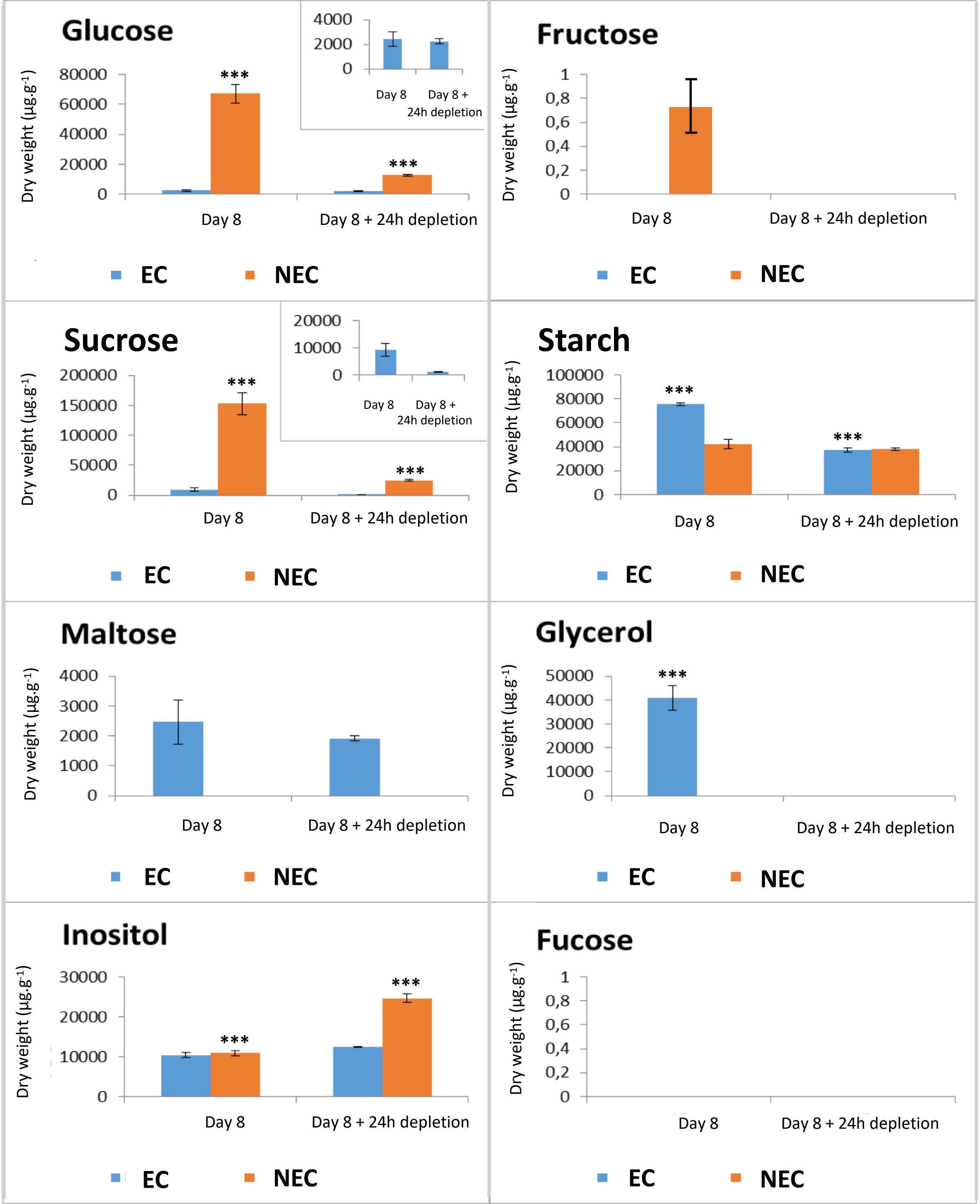

Figure S6. Shifts in concentration of soluble metabolites in embryogenic cells (EC) and non-embryogenic cells (NEC) grown under two conditions: 8 days of culture (D8) and 8 days of culture followed by 24 hours of depletion (D8+24h). The soluble metabolites were measured by ^{1}H NMR. Starch was quantitated by an enzymatic method. The presented results correspond to the mean values of three independent biological replicates (±SEM). The asterisks denote statistically significant differences (ANOVA with Tukey's multiple comparison test; p<0.05).

Figure S6.1

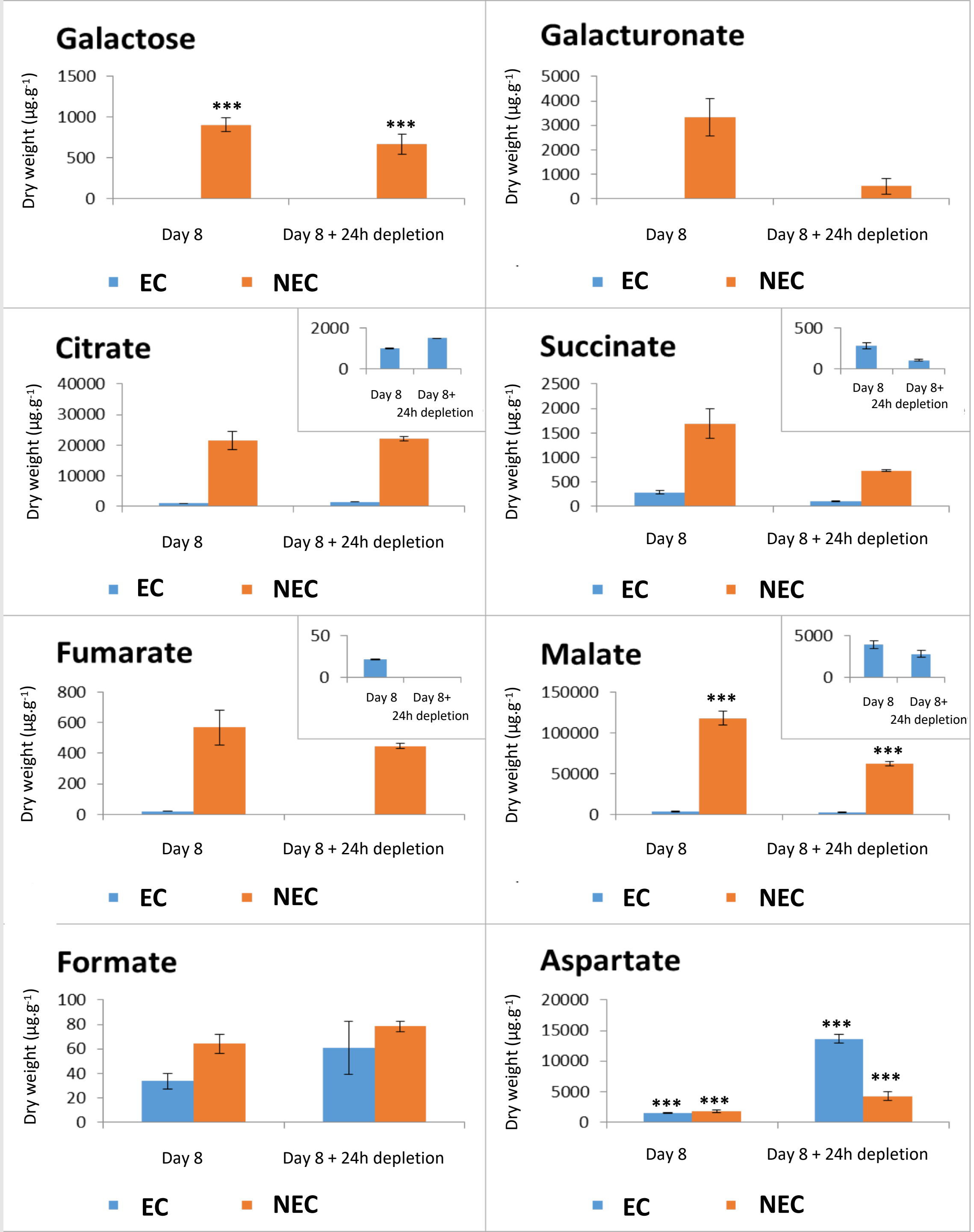

Galactose
Galacturonate
Citrate
Succinate
Fumarate
Malate
Formate
Aspartate
Dry weight (µg.g-1)
Day 8
Day 8 + 24h depletion
Day 8+
24h depletion
EC
NEC
***

Figure S6.2

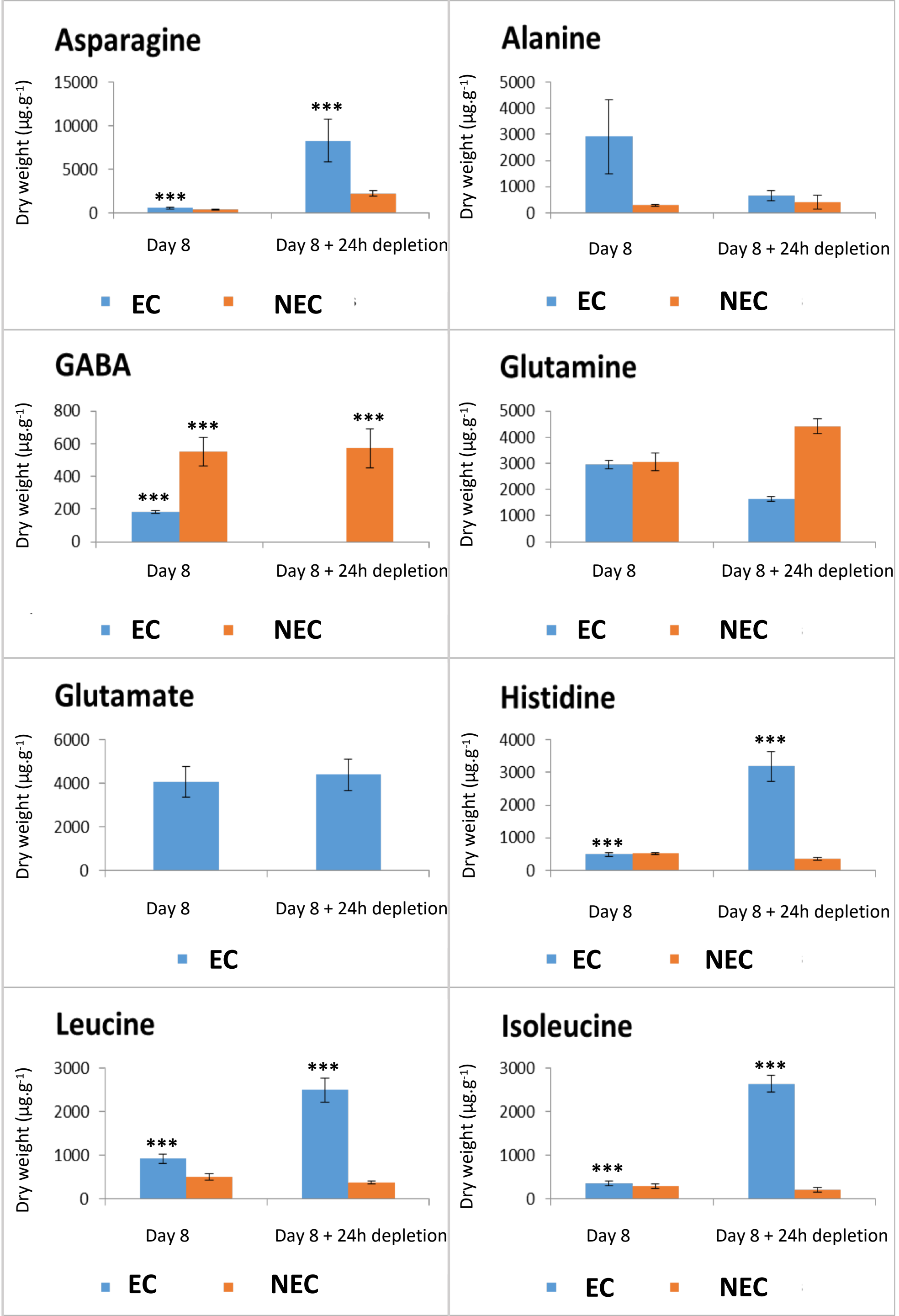
Asparagine
Alanine
GABA
Glutamine
Glutamate
Histidine
Leucine
Isoleucine
Dry weight (µg.g-1)
Day 8
Day 8 + 24h depletion
***
EC
NEC

Figure S6.3

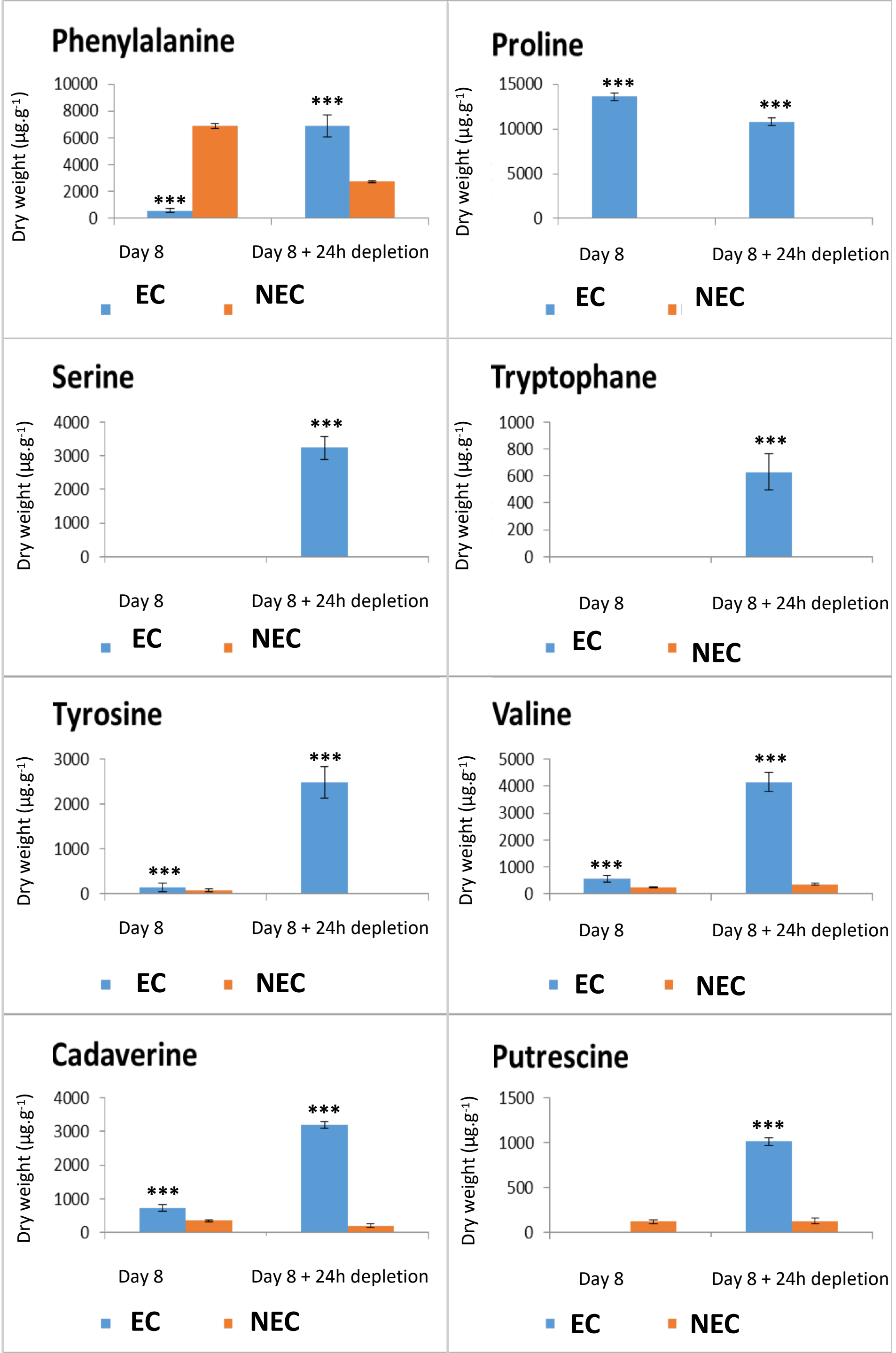
Phenylalanine
Proline
Serine
Tryptophane
Tyrosine
Valine
Cadaverine
Putrescine
Dry weight (µg.g-1)
Day 8
Day 8 + 24h depletion
EC
NEC
***

Figure S6.4

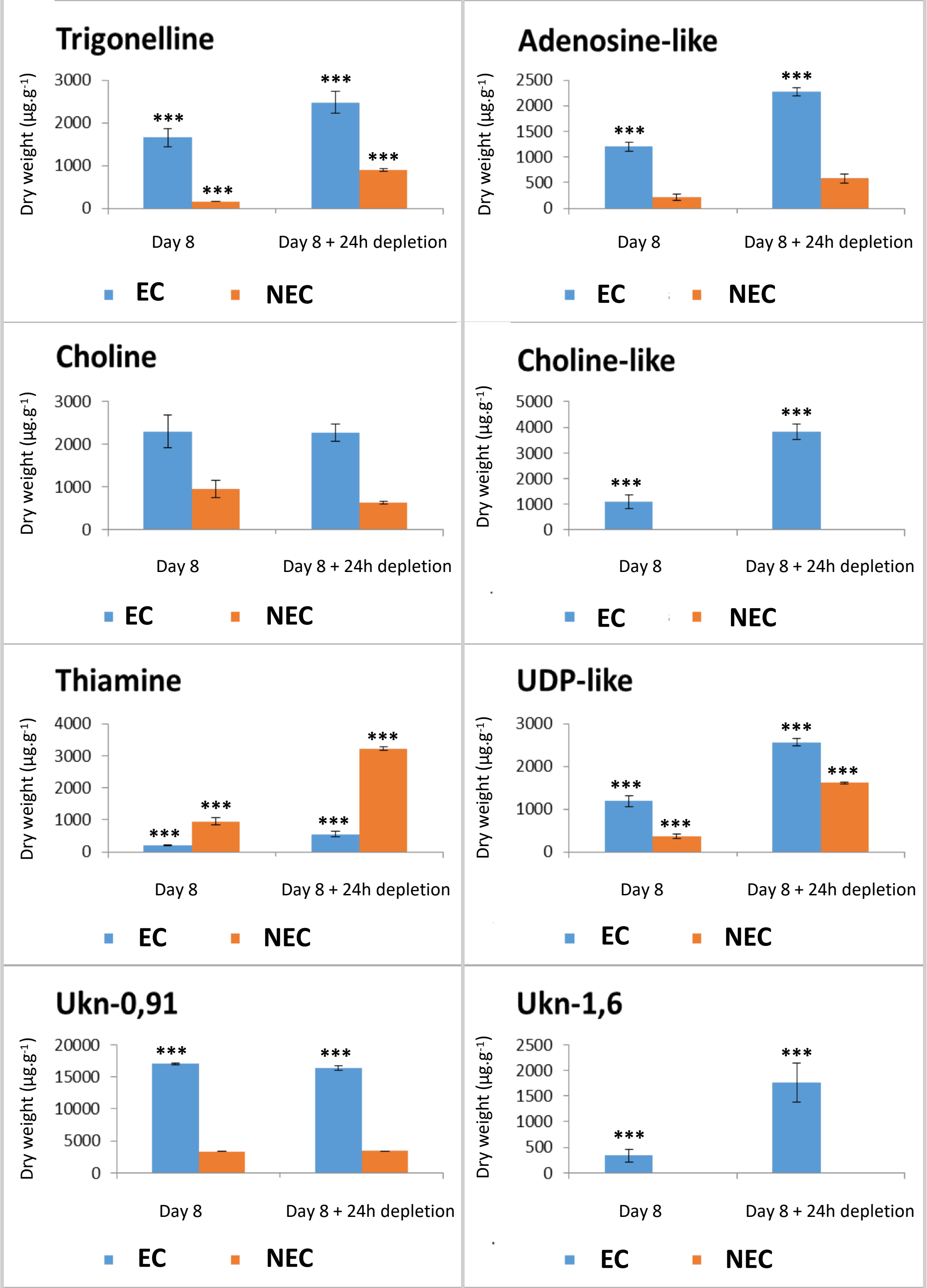

Trigonelline
Dry weight (µg.g-1)
3000
2000
1000
0
***
***
***
***
Day 8
Day 8 + 24h depletion
EC
NEC
Adenosine-like
Dry weight (µg.g-1)
2500
2000
1500
1000
500
0
***
***
Day 8
Day 8 + 24h depletion
EC
NEC
Choline
Dry weight (µg.g-1)
3000
2000
1000
0
Day 8
Day 8 + 24h depletion
EC
NEC
Choline-like
Dry weight (µg.g-1)
5000
4000
3000
2000
1000
0
***
***
Day 8
Day 8 + 24h depletion
EC
NEC
Thiamine
Dry weight (µg.g-1)
4000
3000
2000
1000
0
***
***
***
***
Day 8
Day 8 + 24h depletion
EC
NEC
UDP-like
Dry weight (µg.g-1)
3000
2000
1000
0
***
***
***
***
Day 8
Day 8 + 24h depletion
EC
NEC
Ukn-0,91
Dry weight (µg.g-1)
20000
15000
10000
5000
0
***
***
Day 8
Day 8 + 24h depletion
EC
NEC
Ukn-1,6
Dry weight (µg.g-1)
2500
2000
1500
1000
500
0
***
***
Day 8
Day 8 + 24h depletion
EC
NEC

Figure S6.5

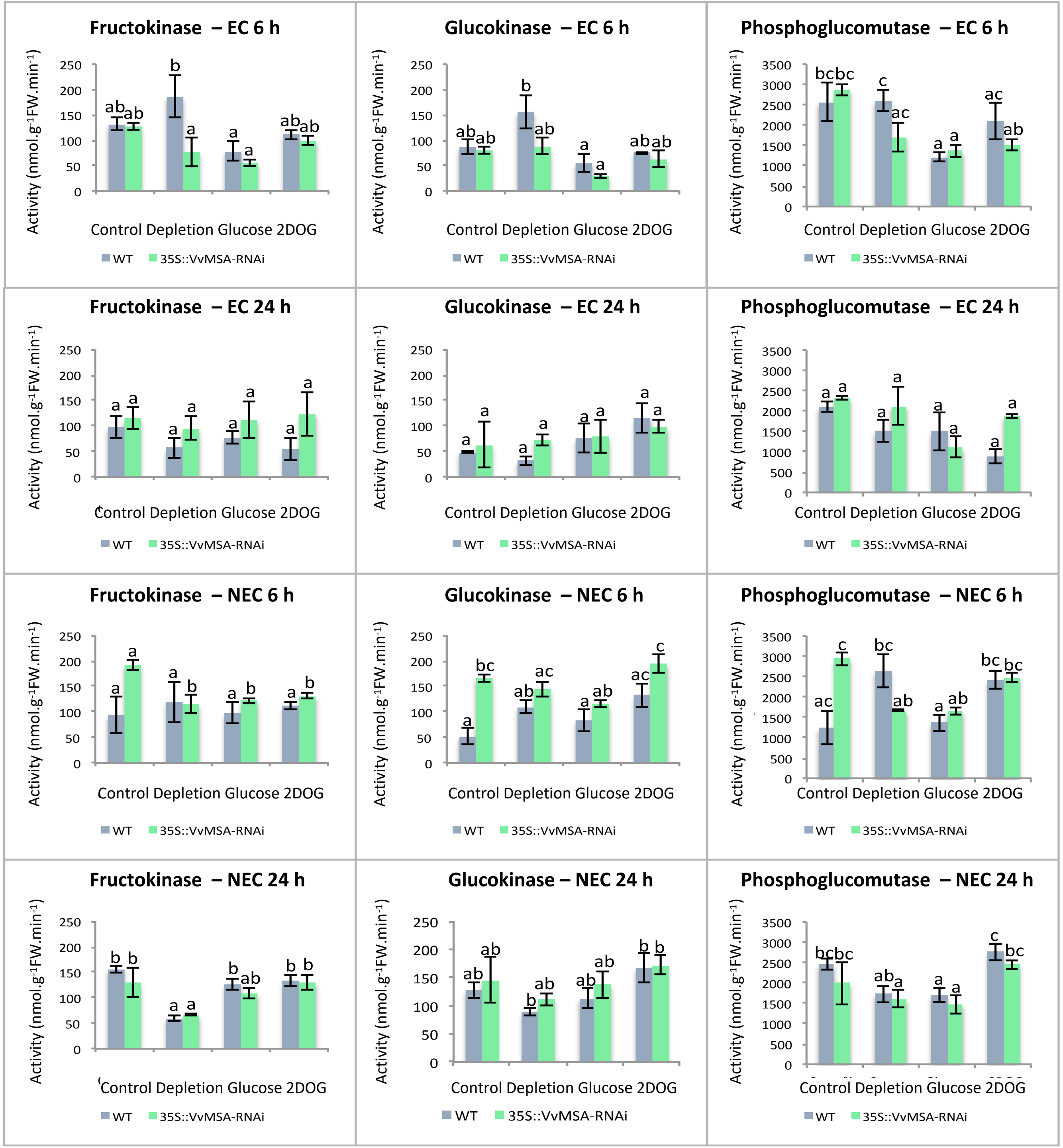
Fructokinase – EC 6 h
Glucokinase – EC 6 h
Phosphoglucomutase – EC 6 h
Fructokinase – EC 24 h
Glucokinase – EC 24 h
Phosphoglucomutase – EC 24 h
Fructokinase – NEC 6 h
Glucokinase – NEC 6 h
Phosphoglucomutase – NEC 6 h
Fructokinase – NEC 24 h
Glucokinase – NEC 24 h
Phosphoglucomutase – NEC 24 h
Activity (nmol.g-1FW.min-1)
Control Depletion Glucose 2DOG
WT
35S::VvMSA-RNAi

Figure S7.1

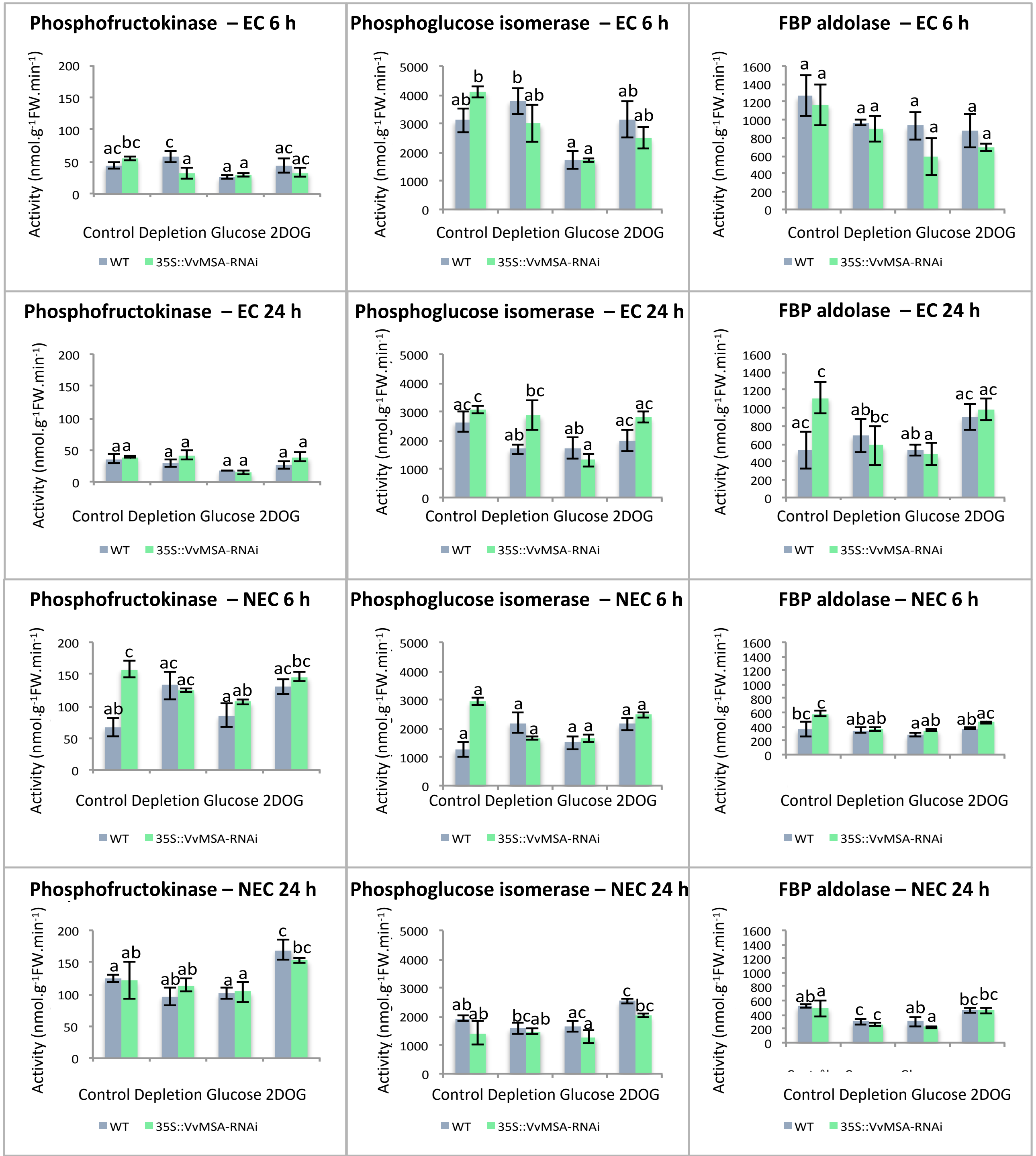

Phosphofructokinase – EC 6 h
Phosphoglucose isomerase – EC 6 h
FBP aldolase – EC 6 h
Phosphofructokinase – EC 24 h
Phosphoglucose isomerase – EC 24 h
FBP aldolase – EC 24 h
Phosphofructokinase – NEC 6 h
Phosphoglucose isomerase – NEC 6 h
FBP aldolase – NEC 6 h
Phosphofructokinase – NEC 24 h
Phosphoglucose isomerase – NEC 24 h
FBP aldolase – NEC 24 h
Activity (nmol.g-1FW.min-1)
Control Depletion Glucose 2DOG
WT
35S::VvMSA-RNAi

Figure S7.2

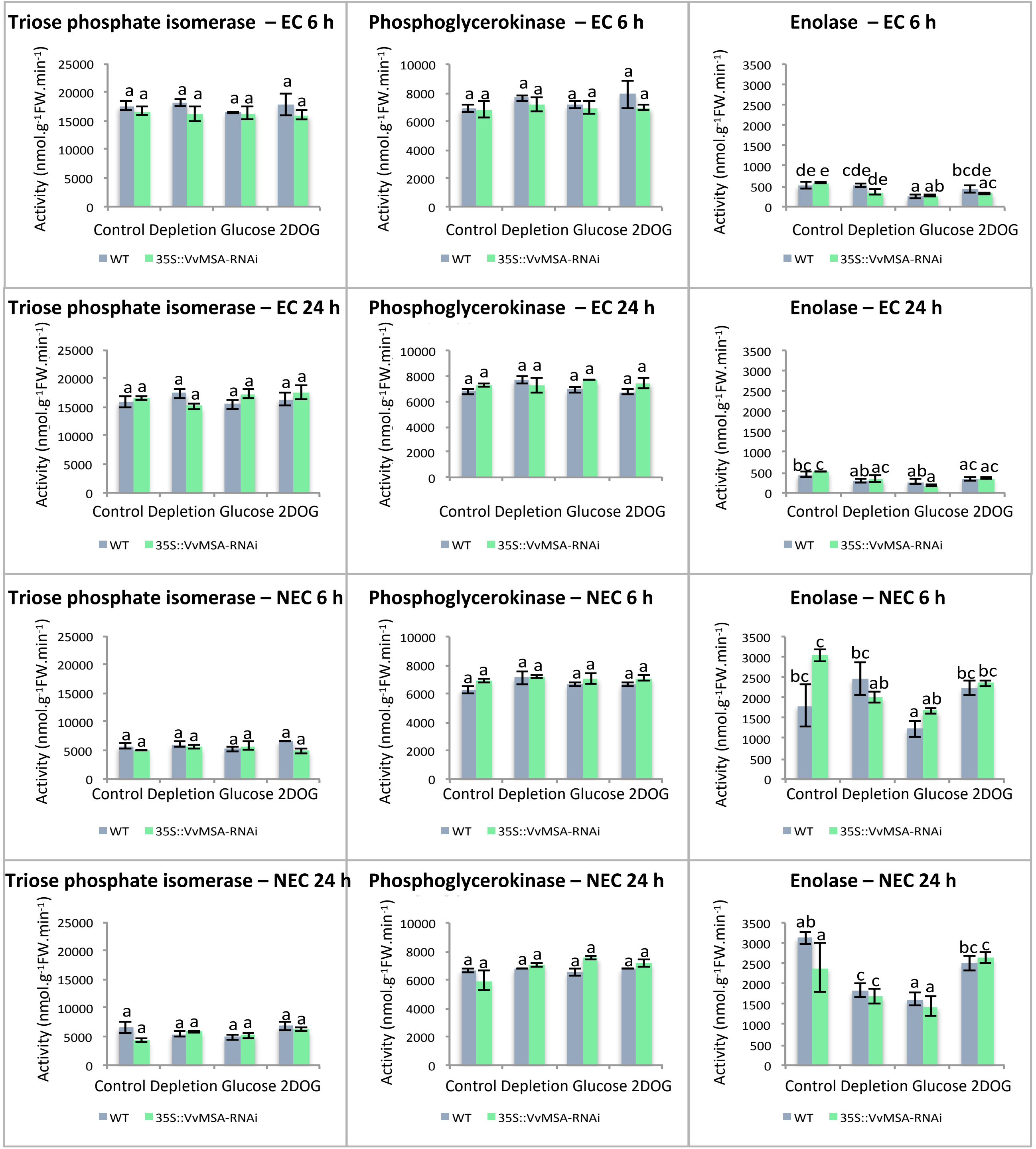
Triose phosphate isomerase – EC 6 h
Phosphoglycerokinase – EC 6 h
Enolase – EC 6 h
Triose phosphate isomerase – EC 24 h
Phosphoglycerokinase – EC 24 h
Enolase – EC 24 h
Triose phosphate isomerase – NEC 6 h
Phosphoglycerokinase – NEC 6 h
Enolase – NEC 6 h
Triose phosphate isomerase – NEC 24 h
Phosphoglycerokinase – NEC 24 h
Enolase – NEC 24 h
Activity (nmol.g-1FW.min-1)
Control Depletion Glucose 2DOG
WT
35S::VvMSA-RNAi

Figure S7.3

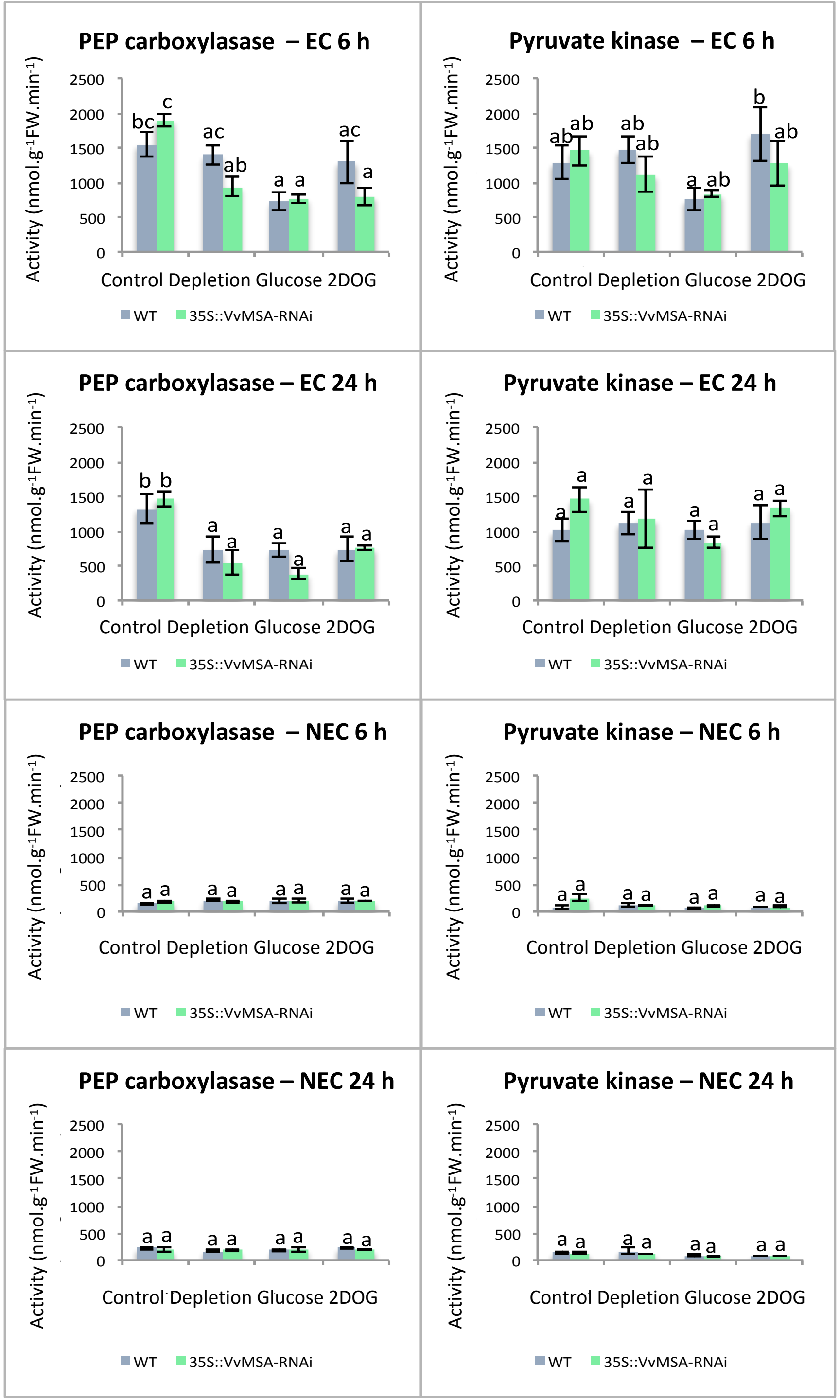

Figure S7. Activities of glycolysis-related enzymes in embryogenic (EC) and non-embryogenic (NEC) wild-type and *35S::VvMSA*-RNAi cells. Studied conditions: Control – cells that were transfected in the complete GM^{+} culture medium or the Gb medium for 30h or 48h; Depletion - cells that were transfected in the minimal GM° culture medium for 30h or 48h; Glucose and 2DOG - cells that were transfected in the minimal GM° culture medium for 24h and treated with 10 mM glucose or 0.9 mM 2DOG for additional time periods of 6 or 24 hours. Three independent biological repetitions were carried out for each of the studied conditions, with at least three technical replicates per biological repetition (mean±SEM).The different letters denote the groups that were found significantly different by ANOVA and Tukey's HSD test (p<0.05).

Figure S7.4

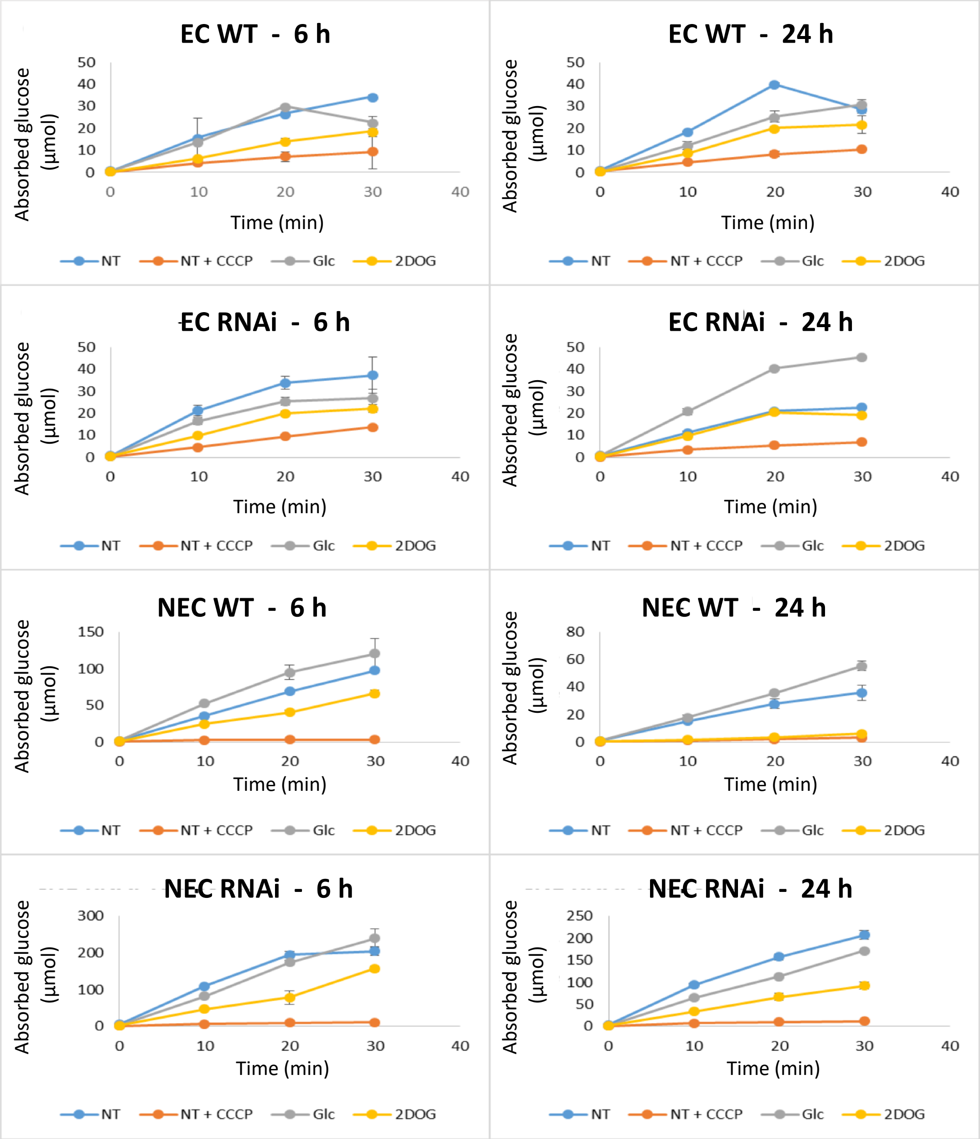

Figure S8. Glucose absorption activity in embryogenic cells (EC) and non-embryogenic (NEC) wild-type and VvMSA-RNAi cells. The embryogenic cells and the non-embryogenic cells were collected after 8 days of subculture and incubated for additional 24 hours in their respective minimal media, *i.e.* GM° minimal (EC) and Gb minimal (NEC). Sugar effectors were added afterwards and the treatment of EC was extended for 6 hours, while that of NEC for 24 hours. In order to estimate the part of passive transport in non treated cells CCCP was added at 20 µM final concentration ten minutes before the addition of radiolabeled glucose. After the addition of the mixture of radiolabeled glucose/glucose (0.2 mM Ci.mL^{-1}/0.1µ Ci.mL^{-1}) 750 µl aliquotes of cell suspension were collected at 0, 10, 20 and 30 minute. The quantity of absorbed glucose (µmol) was evaluated by measurement of ^{14}C-glucose uptake. The presented results correspond to the mean±SEM of three technical replicates of two independent biological repetitions.

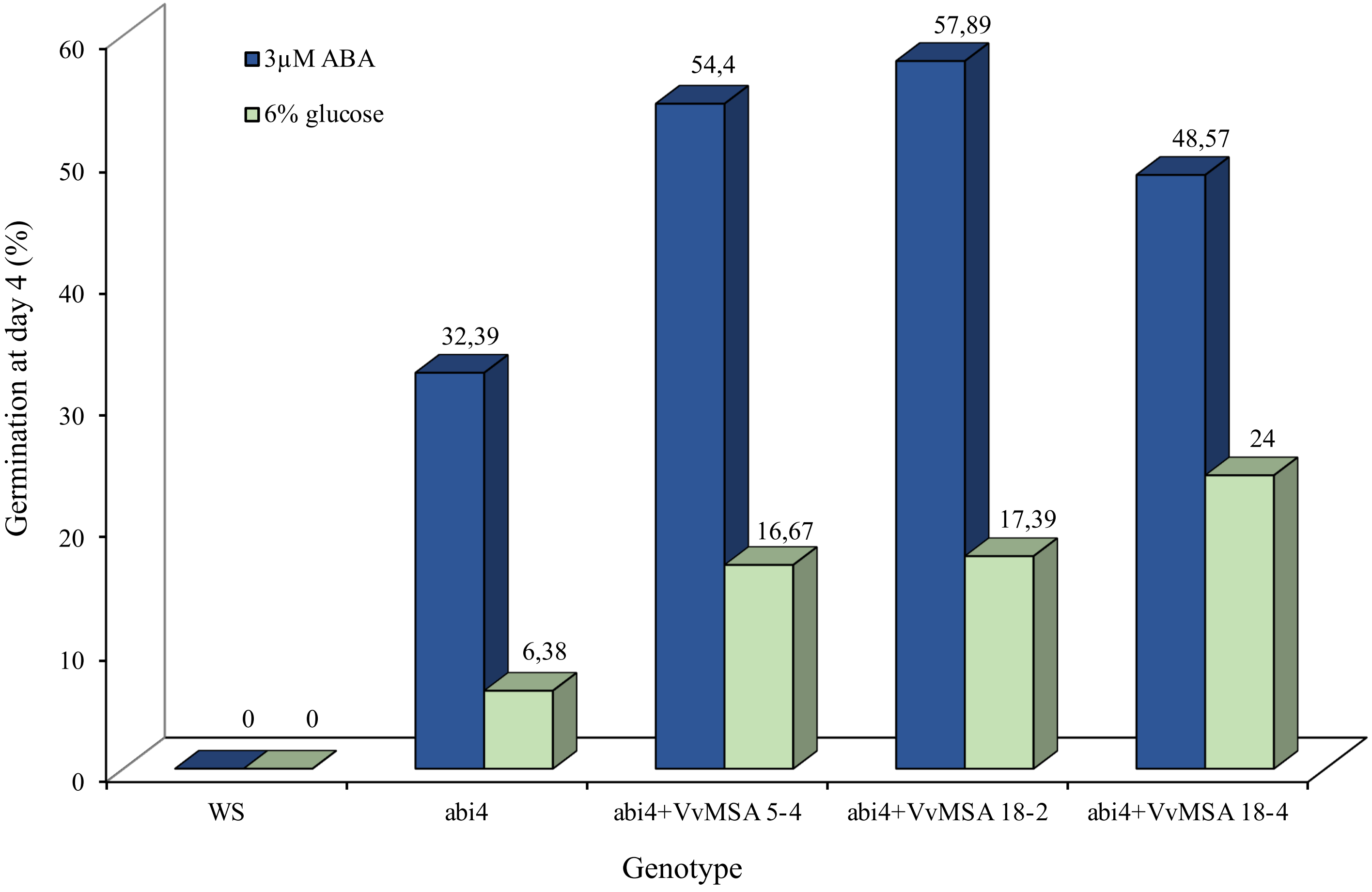

Figure S9. Germination resistance of WT, *abi4* mutant and T3 homozygous *abi4* transformants overexpressing VvMSA to 3 µM ABA and 6% glucose.

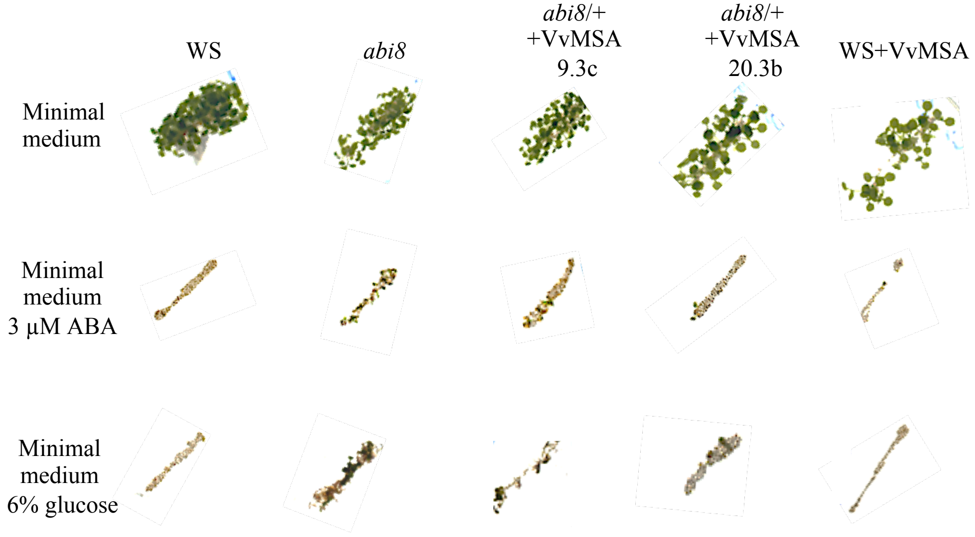

Figure S10. Phenotype of seven-days old germinations of wild genotype WS, abi8/+ mutant and T3 homozygous transformants of abi8/+ and WS overexpressing VvMSA. Seeds were plated on minimal medium as control of their germination capacity, as well as on minimal medium complemented with either 3 µM ABA or 6% glucose.

Table S1. Multifactorial ANOVA analysis of activities of glycolysis-related enzymes in embryogenic and non-embryogenic cells

Embryogenic cells

Enzyme	Genotype	Treatment	Time	Genotype x Time	Genotype x Treatment	Time x Treatment	Genotype x Time x Treatment
Glucokinase						***	
Fructokinase		***	**	***			
Phosphofructokinase		***		*			**
Phosphoglucomutase		***	**	*			
Phosphoglucose isomerase		*	*	*			*
FDP aldolase							
Triose phosphate-isomerase							
Phosphoglycerokinase							
Enolase		***	**				
Pyruvate kinase		**					
PEP carboxylase		***	***				

Non-embryogenic cells

Enzyme	Genotype	Treatment	Time	Genotype x Time	Genotype x Treatment	Time x Treatment	Genotype x Time x Treatment
Glucokinase	***	***					
Fructokinase		***	*			**	
Phosphofructokinase	*	***				*	
Phosphoglucomutase		***					*
Phosphoglucose isomerase	***	***					
FDP aldolase		***					
Triose phosphate-isomerase							
Phosphoglycerokinase	**						
Enolase		***				*	*
Pyruvate kinase		***					
PEP carboxylase							

Analyzed factors: genotype (wild-type and *35S::VvMSA*-RNAi cells), mode of treatment (control, depletion, glucose and 2DOG), time of treatment (6 or 24 hours), as well as interaction between all factors.

Control - cells transfected in complete GM^{+} culture medium or in Gb ANA/BAP medium for 30h or 48h; Depletion - cells transfected in the minimal GM° culture medium for 30h or 48h; Glucose and 2DOG - cells transfected in the minimal GM° culture medium for 24h and treated with 10 mM glucose or 0.9 mM 2DOG for additional time periods of 6 or 24 hours.

Three independent biological repetitions were carried out for each of the studied conditions, with at least three technical replicates per biological repetition.

Level of statistical significance (ANOVA): * - $p < 0.05$; ** $p < 0.01$; *** - $p < 0.001$.
